# Impact of Information and Communication Technologies on Nursing Care: Results of an Overview of Systematic Reviews

**DOI:** 10.2196/jmir.6686

**Published:** 2017-04-25

**Authors:** Geneviève Rouleau, Marie-Pierre Gagnon, José Côté, Julie Payne-Gagnon, Emilie Hudson, Carl-Ardy Dubois

**Affiliations:** ^1^ Faculty of Nursing Sciences Université Laval Quebec, QC Canada; ^2^ Research Center of the Centre Hospitalier de l’Université de Montréal Research Chair in Innovative Nursing Practices Montreal, QC Canada; ^3^ Research Centre of the Centre Hospitalier Universitaire de Québec-Université Laval Quebec, QC Canada; ^4^ Faculty of Nursing Sciences Université de Montréal Montreal, QC Canada; ^5^ School of Nursing McGill University Montreal, QC Canada

**Keywords:** information and communication technology, eHealth, telehealth, nursing care, review, overview of systematic review

## Abstract

**Background:**

Information and communication technologies (ICTs) are becoming an impetus for quality health care delivery by nurses. The use of ICTs by nurses can impact their practice, modifying the ways in which they plan, provide, document, and review clinical care.

**Objective:**

An overview of systematic reviews was conducted to develop a broad picture of the dimensions and indicators of nursing care that have the potential to be influenced by the use of ICTs.

**Methods:**

Quantitative, mixed-method, and qualitative reviews that aimed to evaluate the influence of four eHealth domains (eg, management, computerized decision support systems [CDSSs], communication, and information systems) on nursing care were included. We used the nursing care performance framework (NCPF) as an extraction grid and analytical tool. This model illustrates how the interplay between nursing resources and the nursing services can produce changes in patient conditions. The primary outcomes included nurses’ practice environment, nursing processes, professional satisfaction, and nursing-sensitive outcomes. The secondary outcomes included satisfaction or dissatisfaction with ICTs according to nurses’ and patients’ perspectives. Reviews published in English, French, or Spanish from January 1, 1995 to January 15, 2015, were considered.

**Results:**

A total of 5515 titles or abstracts were assessed for eligibility and full-text papers of 72 articles were retrieved for detailed evaluation. It was found that 22 reviews published between 2002 and 2015 met the eligibility criteria. Many nursing care themes (ie, indicators) were influenced by the use of ICTs, including time management; time spent on patient care; documentation time; information quality and access; quality of documentation; knowledge updating and utilization; nurse autonomy; intra and interprofessional collaboration; nurses’ competencies and skills; nurse-patient relationship; assessment, care planning, and evaluation; teaching of patients and families; communication and care coordination; perspectives of the quality of care provided; nurses and patients satisfaction or dissatisfaction with ICTs; patient comfort and quality of life related to care; empowerment; and functional status.

**Conclusions:**

The findings led to the identification of 19 indicators related to nursing care that are impacted by the use of ICTs. To the best of our knowledge, this was the first attempt to apply NCPF in the ICTs’ context. This broad representation could be kept in mind when it will be the time to plan and to implement emerging ICTs in health care settings.

**Trial Registration:**

PROSPERO International Prospective Register of Systematic Reviews: CRD42014014762; http://www.crd.york.ac.uk/PROSPERO/display_record.asp?ID=CRD42014014762 (Archived by WebCite at http://www.webcitation.org/6pIhMLBZh)

## Introduction

### Background

The use of information and communication technologies (ICTs) for health, referred to as eHealth [1,2] represent a means to support health care delivery [3]. These technologies change how nurses plan, deliver, document, and review clinical care; this will only continue as technology advances. The processes whereby nurses receive and review diagnostic information, make clinical decisions, communicate and socialize with patients and their relatives, and implement clinical interventions will be fundamentally modified with further integration of ICTs into nursing practice [4,5].

There is a wide range of ICTs used for supporting and providing health care. Mair et al [6] suggested four general domains of eHealth that include a variety of ICTs: management systems, communication systems, computerized decision support systems (CDSSs), and information systems. Management systems allow for the acquisition, storage, transmission, and display of administrative or clinical activities related to patients, such as electronic health records (EHRs) or electronic medical records (EMRs). Communication systems can be used for diagnostic, management, counseling, educational, or support purposes. They can be implemented to facilitate communication between health professionals or between health professionals and patients. There are a wide range of communication systems, varying from email and mobile phones to telemedicine and telecare systems. CDSSs are automated systems accessible from various devices, such as computer, mobile phone, or personal digital assistants (PDAs). They support decision-making for health professionals and assist them in practicing within clinical guidelines and care pathways. Information systems, such as Web-based resources and eHealth portals, refer to the use of Internet technology to access health-related information sources.

To support complex and diversified practices and interventions in nursing, myriad ICTs can be adopted, though not without challenges. Some ICTs, such as EHRs and computerized nursing care plans, facilitate access to patient information and help to document and plan nursing care [7]. However, with the use of these technologies, nurses are expected to change the way they document patient care by shifting from paper-based records to electronic systems. The features (eg, copy and paste, electronic interface, drop-down menus) of electronic nursing documentation may affect critical thinking and accuracy of documentation [8]. Telehealth technologies are another example, which include a wide range of ICTs such as remote patient monitoring, videoconferencing, and computer-mediated communications [9]. In the case of remote patient monitoring (telemonitoring), nurses must be able to process a large quantity of data from the system (eg, vital signs, symptoms) and then use clinical decision skills to respond properly to each patient’s condition [10]. In order to discern cues within the interactions via technological modalities, specific communication skills remain essential, that is, active listening, facilitating conversation, questioning, redirecting, and verifying [11-13].

ICTs are becoming an impetus for quality health care delivery by nurses. It is thus relevant to study the role of nurses in the clinical use of ICTs [3] as well as the impact of ICTs on nursing practices [14]. The use of any type of ICT to provide direct or indirect care to patients may transform nurses’ day-to-day practice [3]. In some systematic reviews, different types of ICTs have been reviewed, for instance, EHRs [15], nursing computerized records systems [16], or CDSSs [17]. In general, nursing practice or nursing care was not well-defined in those reviews, and there was no conceptual framework enabling reflection on the way ICTs could influence indicators of nursing care. To overcome this gap, we used a broad and comprehensive conceptualization of nursing care based on the nursing care performance framework (NCPF) [18] to embrace a multidimensional perspective of nursing care. The NCPF is composed of three distinct but interrelated subsystems: nursing resources, nursing services, and patients’ conditions. It is defined as “the capacity demonstrated by an organization or an organizational unit to acquire the needed nursing resources and use them in a sustainable manner to produce nursing services that effectively improve patients’ conditions ([18], p.6).”

However, an integrated body of knowledge was lacking with respect to the effects of ICTs on nursing care, because of the heterogeneity of ICTs used in the literature as well as the poor conceptualization of nursing care. We conducted an overview of systematic reviews to develop a broad picture of the indicators of nursing care that have the potential to be enhanced or constrained by the use of ICTs. The use of an overview is an interesting starting point from which to compare and contrast outcomes of separate reviews [19] regarding the positive, negative, and neutral effects of ICTs on nursing care.

### Objectives

We conducted an overview of systematic reviews to systematically summarize the evidence that comes from qualitative, quantitative, and mixed-method systematic reviews regarding the effects of ICTs on nursing care.

### Nursing Care Performance Framework

In order to illustrate how ICTs interventions influence nursing care and impact health outcomes, an organizational model was used [18]. The NCPF represents a synthesis of the most recent developments in the field and is part of leading initiatives aiming to conceptualize nursing care performance. Conceptualization of nursing care performance is based on a system perspective that builds on system theory [20], Donabedian’s earlier works on health care organization [21], and Parsons’ theory of social action [22].

**Figure 1:**
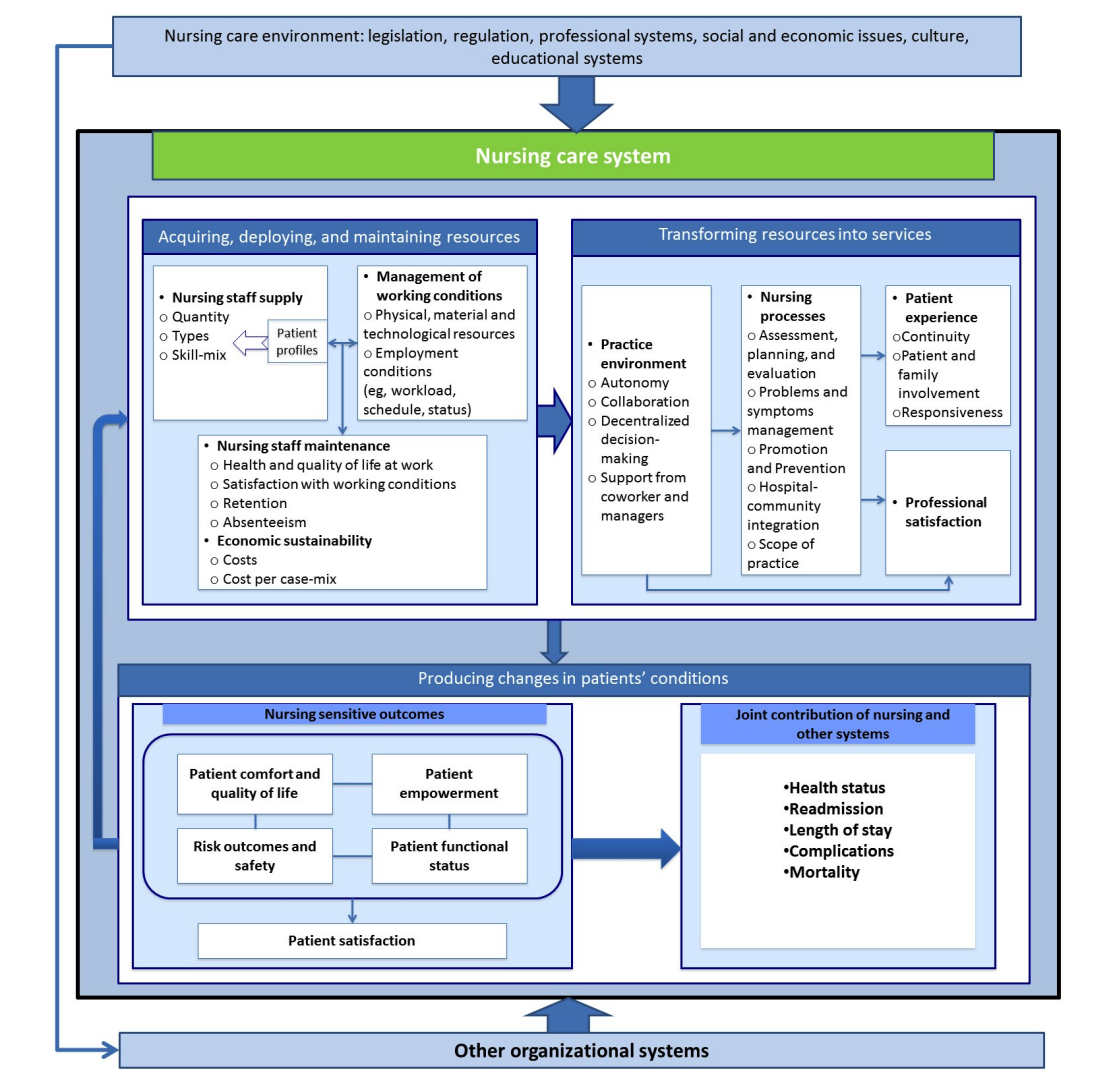
Nursing care performance framework.

This model, illustrated in Figure 1, is composed of 14 dimensions and 51 indicators and shows how the interplay of three nursing subsystems (resources, processes or services, and patients’ outcomes) can operate to achieve three key functions: (1) acquiring, deploying, and maintaining nursing resources; (2) transforming nursing resources into nursing services; and (3) producing changes in patients’ conditions in response to the nursing services provided (“nursing-sensitive outcomes”). The first function refers to the human and material resources needed to provide effective nursing care, such as nursing staff supply, working conditions, staff maintenance, and economic sustainability. The second function encompasses nurses’ practice environments (eg, nurse autonomy; collaboration), nursing processes (eg, assessment, care planning, and evaluation; problems and symptom management), nurses’ professional satisfaction, and patient experience. The desirable end result of the interactions between nursing staff and nursing processes is to improve patients’ conditions. The third function is then described as the positive changes that can be detected among patients (also called “nursing-sensitive outcomes”).

The 51 indicators capture the content currently supported by the scientific literature and cover all major areas of nursing care performance. More than a simple list of indicators, the NCPF provides an integrative and systemic framework that has been used in recent studies to analyze various dimensions of nursing care [23,24]. The NCPF has been used, for example, to structure a scoping review undertaken to identify indicators that are sensitive to ambulatory nursing [23]. The results showed the capacity of NCPF to be extended and applied to ambulatory nursing care and furthermore, five new indicators have been added to the framework. The authors of the NCPF have suggested that further studies should be conducted to assess the implementation of the framework in different contexts of nursing care [18]. This overview constitutes a first attempt to use and apply the NCPF to structure and analyze the indicators of nursing care that are influenced by ICTs. We expect that using the NCPF will confirm existing indicators, add new indicators specific to the context of ICTs, and eventually modify existing indicators.

In this overview, our main interest was to extract data related to nurses. For instance, if results of a systematic review were exclusively on patient outcomes without describing nursing resources, services, or processes, the review was excluded. However, we considered nursing sensitive outcomes (ie, patients’ outcomes) as long as they could be related to ICTs use by nurses.

## Methods

### Overview and Eligibility Criteria

The protocol of this overview has been registered on PROSPERO (CRD42014014762) and published elsewhere [25]. We followed the Cochrane Collaboration methodology [26] and other relevant works in this domain [19,27] to develop the overview. The scope was formulated using PICOS (participants, interventions, comparisons, outcomes, study design) [28,29]. All types of qualitative, mixed-method, and quantitative reviews, published in French, English, or Spanish from January 1, 1995 and that aimed to evaluate the influence of ICTs (four eHealth domains) used by nurses on nursing care were eligible. The inclusion of reviews using multiple methodological approaches is justified by the possibility of broadening the understanding of the impact of ICTs on nursing care. The populations of interest were registered nurses (RN), nurses in training, nursing students, or patients receiving care from qualified RN through the medium of ICTs. The interventions targeted were the use of ICTs covered in the four eHealth domains suggested by Mair et al [6]: (1) management systems; (2) communication systems; (3) CDSSs; and (4) information systems. The following ICTs were excluded: (1) nurse management systems, which are purely administrative and designed for the management of human resources and working conditions (eg, scheduling) and nursing staff maintenance (such as retention); (2) educational systems, for example, e-learning initiatives used for the training of nursing students, unless they are applied to direct patient care; and (3) telephone systems, because according to most definitions of ICTs [30,31], they are not digital technologies and cannot support the electronic capture, storage, processing, and exchange of information. Further details of the inclusion criteria for the selection of systematic reviews are described in Table 1.

**Table 1:** Inclusion criteria for the selection of systematic reviews.

Criteria	Description of inclusion criteria
Type of reviews	All types of qualitative, mixed-method, and quantitative reviews that aimed to evaluate the influence of ICTs^a^(four eHealth domains) used by nurses on nursing care, which stated a methodology (a “Methods” section) with explicit eligibility criteria, had systematic research strategies to identify selected reviews and provided a systematic presentation and summary of the characteristics and outcomes of the included reviews [28].
Publication type	Reviews published in French, English, or Spanish from January 1, 1995.
Population	RN^b^, nurses in training, nursing students, or patients receiving care from qualified RN through the medium of ICTs.
Intervention: ICTs covered by four eHealth domains	Four eHealth domains were considered in the overview [6]: management systems, communication systems, computerized decision support systems, and information systems. ICTs embody all digital technologies that support the electronic capture, storage, processing, and exchange of information, in order to promote health, prevent illness, treat disease, manage chronic illness, and so on [30,31].
Management systems	Management systems are computer-based systems used for acquiring, storing, transmitting, and displaying patient administrative or health information from different sources. They can support administrative or clinical activities. Electronic health records (EHRs) and personal health records (PHRs) are examples of management systems.
Communication systems	Telecommunication systems are employed when users are distant in space and/or time. This kind of communication takes place in a synchronous or an asynchronous way, between health professionals, or between health professionals and patients or caregivers. It involves a targeted sharing of information between specific individuals, or individuals who play distinct roles for diagnostic, management, counseling, educational, or support purposes. There are a wide range of communication systems, from email and mobile phones to telemedicine and telecare systems.
Computerized decision support systems (CDSSs)	Refer to an automated computer-based system that aims to support health professionals in practicing within clinical guidelines and care pathways. These systems are usually operated in real-time and involve decision support that comes from artificial intelligence (eg, a software program).
Information systems	Are defined by the use of Internet technology to attain access to different information resources, such as health and lifestyle information. The information remains general, and it is not tailored to specific individual needs. Web-based resources and eHealth portals for retrieving information are some types of information systems.
Comparisons	Usual care, any other ICT, and other types of interventions.
Outcomes	The primary outcomes included nursing resources, nurses’ practice environment, nursing processes or scope of practice, professional satisfaction, and nursing-sensitive outcomes (eg, patient outcomes, such as risk outcomes and safety, patient comfort, and quality of life related to care). The secondary outcomes included nurses’ and patients’ satisfaction or dissatisfaction with ICTs.

^a^ICTs: information and communication technologies.

^b^RN: registered nurse.

### Search Strategy

A medical librarian developed and conducted the search strategies, drawing on other reviews of similar topics and using well-established search filters where appropriate. We searched publications in English, French, or Spanish in the following electronic databases from January 1, 1995: Cochrane Database of Systematic Reviews (until January 15, 2015); Epistemonikos (until December 25, 2014); PubMed (until December 8, 2014); Embase (until January 7, 2015); Web of Science (until January 9, 2015); and Cumulative Index to Nursing and Allied Health Literature (CINAHL) (until December 25, 2014).

Structured search strategies were developed using the thesaurus terms of each database (eg, Medical subject heading (MeSH) for PubMed) and using free text, targeting the “title” and “abstract” fields. The strategies were then adapted to the other databases. The results of each database search were collected in a single reference database, and duplicate citations were removed. The specific search strategies for databases are presented in Multimedia Appendix 1.

### Selection of Reviews

Two reviewers (GR, JPG) independently screened the title and abstract of the papers in order to assess their eligibility. References that did not meet the preestablished inclusion criteria were excluded. Full-text copies of publications were retrieved and were assessed by the same two reviewers. Any discrepancies were resolved through discussion. A third reviewer was available for arbitration when consensus was not reached.

### Data Extraction and Management

Three reviewers (GR, JPG, and EH) were involved in the data extraction and management process. Information on each review was independently extracted by two of the reviewers. Any disagreement arising during the data extraction process was discussed among the two reviewers. The third reviewer was involved in case of disagreement.

Characteristics of included reviews were extracted and summarized: objectives, type of review, number of included studies, search dates, population, setting, eHealth domain, types of general and specific ICTs, examples of included interventions, comparisons, primary and secondary outcomes, review limitations, and authors’ conclusions. A data extraction form was developed based on the NCPF [18] and the dimensions of the actual scope of nursing practice [32]. The data extraction grid was modified during the extraction process by adding dimensions or categories of results. To facilitate teamwork between the three reviewers (GR, JPG, and EH) in performing the data extraction, we used a shared file in Google Sheets. Reviewers communicated with each other through Google Sheets and added comments on the extraction when needed. The three reviewers reviewed the completed data extraction grid to eliminate discrepancies and errors.

### Methodological Quality Assessment of Included Reviews

The three reviewers (GR, JPG, and EH) were involved in the methodological quality assessment of the reviews that met the eligibility criteria, using the assessment of multiple systematic reviews (AMSTAR) tool [33,34]. Two reviewers assessed each review independently, and disagreements were discussed. The third reviewer was available for arbitration when needed. AMSTAR is an 11-item checklist from which reviewers assign one point when the criterion is met. AMSTAR items provide an assessment of methodological criteria such as the comprehensiveness of the search strategy and whether the quality of included studies was evaluated and accounted for [35]. AMSTAR characterizes quality at three levels: 8-11 is high quality (ie, minor or no methodological limitations), 4-7 is medium quality (ie, moderate methodological limitations), and 0-3 is low quality (ie, major methodological limitations) [36].

In this overview, we included different types of systematic reviews, that is, quantitative reviews (randomized and nonrandomized designs), mixed-method synthesis reviews, and qualitative reviews. AMSTAR is used primarily for quantitative reviews using randomized controlled trial (RCT) design. When undertaking an overview, challenges encountered are the assessment of limitations (risk of bias) as well as the quality of evidence in systematic reviews [37,38]. There were no reporting guidelines on assessing methodological quality of mixed-method and qualitative reviews at the time of the overview. We decided to apply AMSTAR to all reviews in order to use the same criteria for quality assessment, although this had limitations (ie, inappropriateness of applying some criteria to mixed-method and qualitative reviews).

### Data Synthesis

A statistical meta-analysis of outcomes was not possible because the included studies were too heterogeneous. We therefore conducted a narrative synthesis, which is defined as an approach of summarizing and explaining outcomes from multiple studies by employing the use of words and text [39]. The core characteristic of a narrative synthesis is the adoption of a “textual approach to the process of synthesis to ‘tell the story’ of the outcomes from the included studies” [39]. We categorized the reviews into subgroups according to the type of intervention and their effects (positive, negative, or no effect) on a specific dimension of nursing care (eg, practice environment, nursing processes, professional satisfaction, and nursing-sensitive outcomes).

## Results

### Description of the Reviews

**Figure 2:**
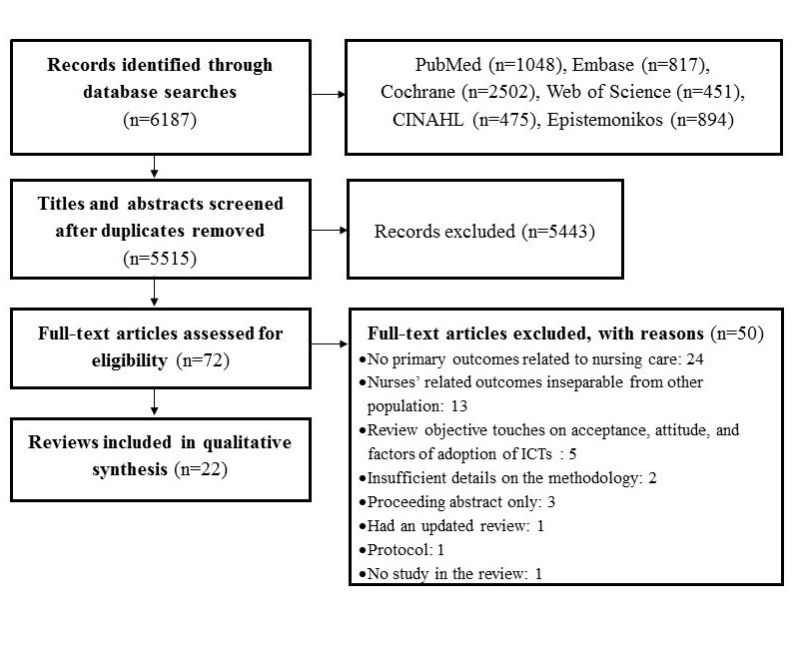
The preferred reporting items for systematic reviews and meta-analyses (PRISMA) study flow diagram. ICT: information and communication technology.

A total of 6187 titles or abstracts were identified. After removing duplicate references, 5515 titles or abstracts were assessed for eligibility. Full-text papers of 72 articles were retrieved for detailed evaluation. It was found that 22 reviews published between 2002 and 2015 met the eligibility criteria. The list of these included reviews is presented in Multimedia Appendix 2. Twelve reviews used a mixed-method synthesis approach, nine used a quantitative approach, and one used a qualitative approach (meta-ethnography). Fifty reviews were mainly excluded because they did not present primary outcomes related to nursing care (n=24), or because outcomes related to nurses were indistinguishable from other populations (n=13). In Multimedia Appendix 3, details are provided regarding the primary reasons for exclusion and the full references of the excluded articles. The preferred reporting items for systematic reviews and meta-analyses (PRISMA) study flow diagram [40] are illustrated in Figure 2 to show the overall process of review selection.

The general characteristics (ie, type of reviews, search dates, target population, and health care settings) of the included reviews are presented in Multimedia Appendix 4. The review objectives, limitations, and main conclusions are synthetized in Multimedia Appendix 5. The eHealth domains covered were management systems (n=14), communication systems (n=7), and CDSSs (n=10). No reviews dealt with information systems. Five reviews included more than one eHealth domain [3,41-44]. Articles reviewing management systems included the following ICTs: electronic medical or health or patient records, computer-based nursing records or computerized nursing care planning, and regional health care information system. The ICTs covered in the communication systems were email, mobile phone, bedside communication tool or bedside terminals, iPod technology to assist in educational conferences, and telemedicine or telehealth with the use of videophone or videoconferencing. The CDSSs covered were medication management technology—e-prescribing, electronic medication administration record systems, computerized provider order entry (CPOE), bar-code medication administration (BCMA) —and PDAs. These eHealth services can be categorized as belonging to more than one domain [6], depending on their components. Details about eHealth domains, examples of included interventions, and comparisons are presented in Multimedia Appendix 6.

### Assessment of Review Quality

The AMSTAR tool was used to assess the methodological quality of all reviews. Four reviews, mostly quantitative ones, were high quality (scores: 8-9); nine were medium quality (scores between 4 and 7), and nine scored low quality (between 0 and 3). AMSTAR scoring for each review is presented in Table 2. We adapted the interpretation of two criteria (#7 and #9) of the AMSTAR tool to assess the quality of mixed-method and qualitative reviews. For the criteria 7—reporting and assessment of scientific quality of the included reviews—we answered “yes” if authors mentioned having assessed and documented quality of quantitative reviews, and if they acknowledged clearly the difficulty of assessing qualitative or mixed-methods reviews. For criteria 9, entailing the inappropriateness of methods used to combine findings, we answered “yes,” based on the decision rules developed by Kitsiou et al [45]: “reviews’ authors made a statement regarding the inappropriateness of pooling data (eg, highlighted issues about heterogeneity or variability between the studies), that is, the authors summarized and synthesized the available evidence narratively according to a defined analysis plan and/or using appropriate qualitative methods and techniques (eg, construction of common rubrics, content analysis, tabulation, groupings, and clustering).” Regarding criteria 10, about the assessment of publication bias, it seems that empirical evidence on this topic in qualitative research is very limited [46]. We presume that this is the same reality regarding the mixed-method reviews.

### Dimensions of Nursing Care That Are Influenced by Information and Communication Technologies

**Figure 3:**
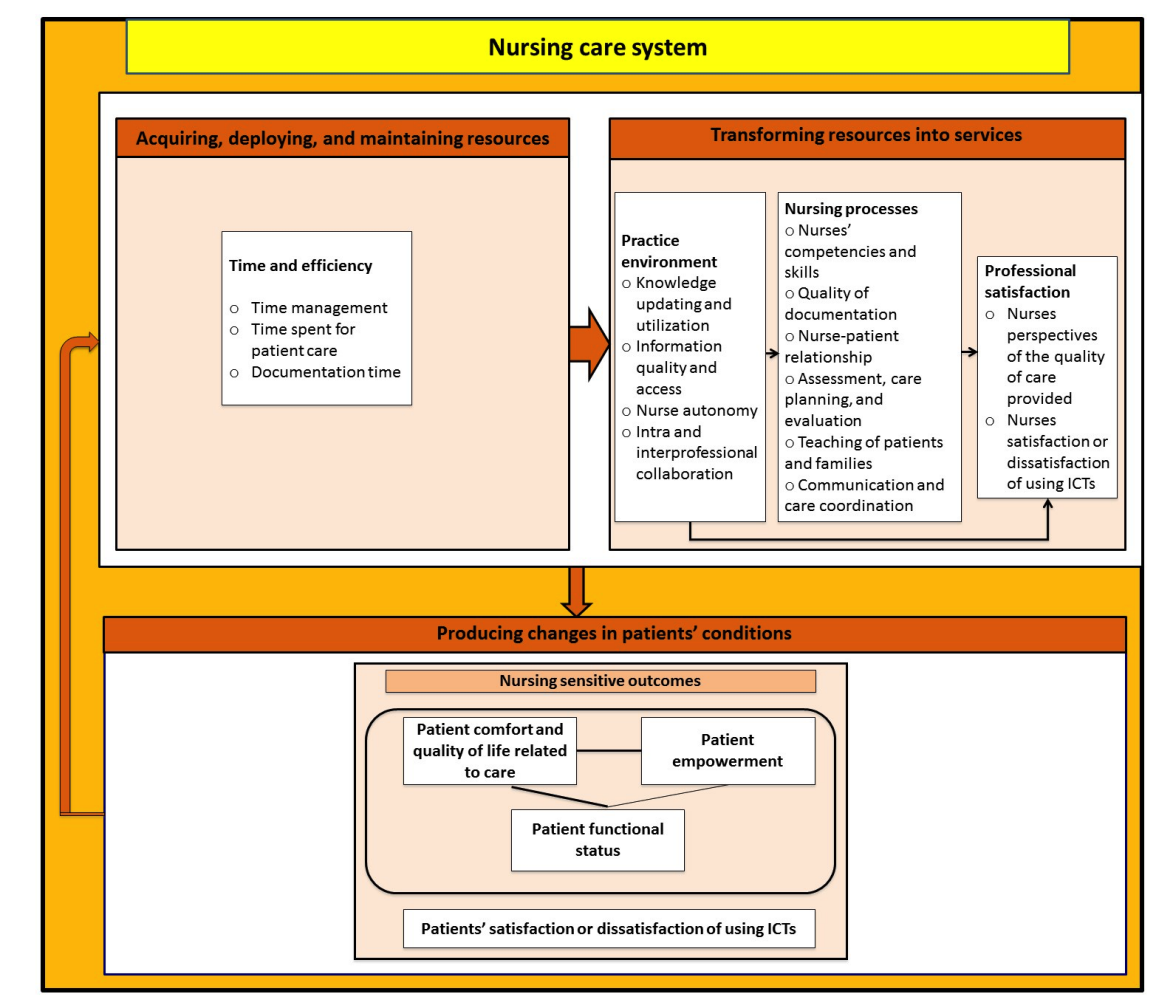
Presentation of results.

The results (see Figure 3) will be presented in association with the NCPF: the function, the dimension, and the theme (which correspond or not to a particular indicator in the framework). Table 3 presents the frequency of extracted data per dimensions, themes, and ICTs.

**Table 2:** Assessment of multiple systematic reviews (AMSTAR) scoring.

References	Type of reviews or designs	AMSTAR score
Free [42]	Quantitative (RCT^a^)	9 (high)
Mador [47]	Quantitative (various designs)	9 (high)
Urquhart [16]	Cochrane review—quantitative (RCT+1 other design)	8 (high)
McKibbon [43]	Mixed	8 (high)
Nieuwlaat [48]	Quantitative (RCT)	7 (medium)
Mickan [49]	Quantitative (RCT)	6 (medium)
Finkelstein [41]	Mixed	6 (medium)
Randell [50]	Quantitative (RCT)	5 (medium)
Georgiou [51]	Quantitative (various designs)	5 (medium)
Dowding [52]	Quantitative (various designs)	5 (medium)
Poissant [53]	Quantitative (various designs)	4 (medium)
Husebo [54]	Mixed (integrative)	4 (medium)
Jones [55]	Mixed (integrative)	4 (medium)
Meißner [56]	Qualitative (meta-ethnography)	3 (low)
Bowles [44]	Mixed	3 (low)
Anderson [17]	Mixed	3 (low)
Maeenpa [57]	Mixed	2 (low)
NGuyen [58]	Mixed	2 (low)
Stevenson [15]	Mixed	2 (low)
Bartoli [59]	Mixed	1 (low)
Carrington [3]	Mixed	1 (low)
Kelley [60]	Mixed (integrative)	0 (low)

^a^RCT: randomized controlled trial.

**Table 3:** Frequency extracted data.

Dimension	Themes (Number of reviews) (Types of eHealth domain)	Positive effects of ICTs^a^	Negative effects of ICTs	No effect	Total
**Time and efficiency**		20	17	7	44
	Time management (4) (MS^b^, CS^c^, CDSS^d^)	2	1	1	4
	Time spent for patient care (7) (MS, CS, CDSS)	4	5	3	12
	Documentation time (7) (MS^e^)	14	11	3	28
**Nurses’ practice environment**		19	5	1	25
	Knowledge updating and utilization (3) (CS, CDSS)	3	0	1	4
	Information quality and access (5) (MS^f^, CDSS)	11	2	0	13
	Nurse autonomy (1) (CS^e^)	1	0	0	1
	Intra and interprofessional collaboration (6) (MS^f^, CS, CDSS)	4	3	0	7
**Nursing processes**		30	12	3	45
	Nurses competencies-skills (4) (MS, CDSS)	9	1	1	11
	Nurse-patient relationship (3) (CS^e^)	4	0	0	4
	Quality of documentation (7) (MS^f^, CS)	6	4	1	11
	Assessment, care planning, and evaluation (10) (MS, CS, CDSS)	13	8	2	23
	Teaching of patients and families (4) (CS^f^, CDSS)	5	0	0	5
	Communication and care coordination (2) (CS, MS)	2	0	0	2
**Professional satisfaction**		29	18	1	48
	Nurses’ perspectives of the quality of care provided (6) (MS, CS, CDSS)	15	2	0	17
	Satisfaction or dissatisfaction of nurses using ICTs (10) MS, CS, CDSS)	14	16	1	31
**Nursing sensitive outcomes**		28	5	5	38
	Patient comfort and quality of life related to care (7) (CS, CDSS)	7	0	1	8
	Empowerment (4) (CS^f^, MS)	6	0	1	7
	Functional status (3) (CS^e^)	3	0	1	4
	Satisfaction or dissatisfaction of patients using ICTs (5) (CS, MS)	12	5	2	19

^a^ICTs: information and communication technologies.

^b^MS: management systems.

^c^CS: communications systems.

^d^CDSSs: computerized decision support systems.

^e^One eHealth domain covered exclusively a particular theme.

^f^Majority of one eHealth domain covered a particular theme.

### Function 1: Acquiring, Deploying, and Maintaining Resources

#### Time and Efficiency

Overall, 11 reviews [15,16,43,44,47,48,51,53,56,58,60] had results related to time: time management (time consumed or time saved resulting the use of ICTs); time spent for patient care; and documentation time.

#### Time Management

Four reviews [43,44,48,53] targeting CDSSs, communication, and management systems had findings on “time management” in a general way: one review showed no effect [48], another showed negative effects [43], and two reported positive effects [44,53]. In Nieuwlaat et al’s [48] review, results demonstrated that nurses perceived that conventional care compared with CDSSs were equally time-consuming (no effect). The other review reported that reminder systems were “time-consuming” [43]. The results in the Poissant et al [53] review revealed that the use of EHRs has been shown to reduce the time devoted to the verbal transmission of information at the end-of-shift. Consequently, this caused a change in the workflow, which may have been a strong incentive for nurses to become efficient users of the system. In the Bowles and Baugh [44] review, the effect of telehomecare was also reported positively in terms on “saving time.”

#### Time Spent for Patient Care

Almost one-third of the reviews (7/23) [16,43,47,51,56,58,60] outlined positive [16,47,56,58] and negative effects [43,51,56,60], as well as no effect [43,51,58] of CDSSs, management systems, and communication systems on time spent for patient care. Nurses are sometimes concerned that using electronic nursing documentation or the BCMA for documenting and for administering medication might take away or reduce time for patient care [43,56]. Conversely, other reviews including communication systems (eg, telehomecare) and management systems (eg, EHRs) found that time spent for patient care has significantly improved [16,47,56,58] and particularly, nurses using EHRs spent more time with patients in assessment, education, and communication [58].

#### Documentation Time

Nurse documentation time was reported in seven reviews [15,16,43,47,53,56,60] touching on management systems, such as EHRs, e-prescribing system, and critical care information system (CCIS). Effects of these ICTs on documentation time were mixed within and across the reviews: six reviews demonstrated positive effects [15,16,47,53,56,60], six demonstrated negative effects [15,16,43,47,53,60], and three demonstrated no effect [43,47,60]. Negative results showed that nurses spent more time documenting when they used management systems and the positive results showed the contrary: documentation time diminished with ICTs. The time saved for documenting was sometimes reallocated for patient care and had positive outcome on the improvement of health care [15]. Otherwise, when the task of documenting took much more time, nurses had less time to spend with patients [60].

### Function 2: Transforming Resources into Services

#### Nurses’ Practice Environment

##### Knowledge Updating and Utilization

Three reviews found positive effects [17,44,54] of CDSSs and communication systems on knowledge updating and utilization, whereas one review found no effect [17]. CDSSs are useful tools to increase knowledge and information use, and translate outcomes from research into practice by improving nurses’ compliance with established guidelines [17]. The potential of communication systems (eg, telehomecare or telehealth) to transfer nursing knowledge was also reported [44,54].

##### Information Quality and Access

The eHealth domain that was the most covered in relation to information quality and access was management systems, covered in four reviews [43,57,58,60], followed by CDSSs in two reviews [17,43]. One review documented the improvement of information quality as perceived by doctors and nurses after the implementation of EHRs [58], and the results of five reviews highlighted information access [17,43,57,58,60]. Management systems and CDSSs had positive impact in three reviews [43,57,60] on information access regarding patient issues, clinical data, medication information or profile, and other information (policies, guidelines, drug resources, patient files). Nurse practitioners felt that CDSSs could assist them with patient care when data is easily accessible with the use of the technology [43]. The negative impact were pointed out in two reviews [58,60] that cited the results of the same primary study [61], that is, nurses could not retrieve the information perceived as essential for patient care within the electronic nursing documentation system.

##### Nurse Autonomy

Only one review mentioned nurse autonomy as a positive effect. In this review [59], nurses were expected to handle most cases autonomously and to refer to doctors only in exceptional cases when using the tele-triage system designed to monitor chronic heart failure patients remotely.

##### Intra- and Interprofessional Collaboration

Four reviews highlighted positive [17,42,52,59] effects regarding intra- and interprofessional collaboration, one showed negative effect [43] and one reported no effect [60] with the use of CDSSs [17], communications [42,59], and management systems [43,52,60]. Reviews including CDSSs reported improved communication between members of the interdisciplinary team [17], such as between nurses and surgeons [42], better and more trustworthy relationships between nurses and doctors by using telehomecare systems [59], and more frequent collaboration between members of the health care team when using management systems (ie, clinical dashboards) [52]. In one review, results showed that electronic nursing documentation systems negatively affected collaborative working relationships between nurses and physicians [60].

#### Nursing Processes

##### Nurses’ Competencies and Skills

Four reviews that encompassed CDSSs and management systems showed that they had a positive influence on these domains of nurses’ competencies and skills: decision support or decision-making [17,43,56], observation skills [56], clinical judgment [17,56], and critical thinking [60]. Additionally, due to some features of CDSSs and management systems (eg, readability of data, remote accessibility of data, better quality of patients’ records, presence of reminders, or automatic alerts), these ICTs supported clinical judgment and decision-making [43,56]. Conversely, some features of the ICTs not previously available on paper, such as copy and paste, drop-down menus, and check boxes, affected the nurses’ capacity to employ critical thinking regarding their patients [60]. Finally, the results presented in Anderson and Willson [17] review showed no effect of CDSSs on the knowledge or clinical decision-making of nurses associated with pressure ulcer prevention.

##### Quality of Documentation

Positive effects on documentation quality were highlighted in six reviews [3,15,43,56,58,60]; five on these reviews encompassed management systems. Negative effects were reported in three reviews [15,56,60], and another review documented no effect [41]. Results from the Stevenson et al [15] review: nurses reported that EHRs did not reflect their practice and reported that it was “incapable of capturing much of what they believed was crucial in nursing care.” With regards to psychological care, nurses also reported issues with fitting complex caring practice into systems that are not intended to accommodate it, for example, when providing emotional and psychological support. Since EPRs lack sensitivity, they cannot capture “the being there stuff,” for example, caring for a dying patient by sitting on their bedside and holding their hand. Two reviews [15,56] stated that when the quality of documentation is improved, quality of care and patient safety can be fostered since it allows a complete overview of the patient’s situation [56].

##### Nurse-Patient Relationship

In three reviews, use of communication systems (virtual visits using videophones, telehomecare, telehealth) positively impacted nurse-patient relationship. Reviews mentioned the potential of ICTs to provide a pathway for communication [55], create new types of bonds with patients [44], establish trust through the videoconference system, and create a sense of connection (from the patients’ perspective)[54].

##### Assessment, Care Planning, and Evaluation

Impact of CDSSs, management, and communication systems were mixed, that is, positive effects were mentioned in seven reviews [15,17,41,44,49,55,56], negative effects were mentioned in five reviews [15,16,41,42,54], and no effect was documented in two reviews [16,55]. For example, a handheld computer-based support system for preference-based care planning led to a higher consistency between patient preferences and nursing care plan priorities [17]. An “email intervention” cited in the Finkelstein et al [41] review led to a more comprehensive heart failure and medication adherence assessment by nurses being recorded. EHRs contain templates that guide nurses for assessment and help them identify problems [56]. The mixed review by Stevenson et al [15] revealed negative impact of EHRs regarding poor care plans updates, the difficulty of individualizing care plans within the systems, and the difficulty of capturing a broad picture of the patient within the electronic personal record. Similarly, the Urquhart et al [16] review showed that computerized nursing care planning compared with manual planning led to (1) no effect between groups regarding planning; and (2) negative effects, because planned tasks were not carried out as expected for nurses using ICTs.

##### Teaching of Patients and Families

Four reviews reported teaching benefits: three with the use of communication systems [41,54,55] and one with CDSSs [43]. For example, virtual visits simplified teaching and information sharing with patients and thus became a way to transfer knowledge [54]. Also, patients had clearer instructions on discharge and on their medication administration at home as reported by nurse practitioners [43].

##### Communication and Care Coordination

Two reviews found that communication systems had positive impact on delivering continuous and coordinated care, on the prevention of preventing relapses into poor health [54], and on improving communication about resident care [56].

#### Professional Satisfaction

##### Nurses’ Perspectives of the Quality of Care Provided

In six reviews, positive effects [41,43,44,54-56] of CDSSs, management, and communication systems were reported: improvement of quality of care and patient safety; nurses’ perceptions that BCMA reduce medication errors and improve medication administration processes [43]; and the provision of comprehensive and adaptive care related to the patients’ needs with the help of telehealth used with elders [55]. In four reviews [15,43,56,58], negative results were discussed: EHRs do not improve patient care as perceived by nurses [58]; and patients do not receive necessary care because the quality of residents’ records is lacking [56].

##### Satisfaction or Dissatisfaction of Nurses Using ICTs

The results in ten reviews, targeting the three eHealth domains, found that nurse satisfaction was mixed: nine reviews reported positive effects [17,41,43,44,48,54,56,58,60], eight reported negative effects [15,43,44,48,54,56,58,60], and one reported no effect [43]. Results pertained to overall acceptance of ICTs and their satisfaction was described in general ways, such as “nurses were satisfied with ICTs.” There were also elements associated with ICTs, such as system navigability (eg, complexity, ease of use, user-friendliness, and flexibility), nurses’ attitudes, concerns about patients’ privacy, and perceived benefits or inconveniences. Some nurses found EHRs to be irrelevant for practice [58].

### Function 3: Producing Changes in Patients’ Condition

#### Nursing-Sensitive Outcomes

##### Patient Comfort and Quality of Life Related to Care

The positive effects of CDSSs and communication systems on comfort and quality of life related to care [3,17,41,44,50,54,55] were described in terms of patient outcomes: fewer number of wetting occurrences [17], reduction of malnourished patients [3,50], the reduction of pain and anxiety [44], better quality of life [41], and lower burden related to care [55]. One review reported little improvement on quality of care with the use of telehomecare [44].

##### Empowerment

Four reviews [16,41,44,54] highlighted empowerment as a positive effect of communication systems. One management system showed no effect [16]. Some examples of positive impact include diabetic patients, who felt that the telehomecare empowered them [44] and had positive results in terms of diabetes management with an eHealth application [41]. One review also cited videoconferences for conducting nursing virtual visits as tools to increase patients’ abilities to manage self-care [54].

##### Functional Status

In three reviews [41,54,55], the results regarding the effects of communication systems on functional status (eg, physical, cognitive, psychosocial functional capacity) were discussed in a positive way. Computer use (in a telehealth context) and elders’ self-esteem have been positively associated [55]. In another review [54], the results showed that communication systems (eg, virtual visits using videoconference) decreased loneliness and melancholia, enhanced psychosocial and social activity, and aided memory among home-dwelling elders. In the Finkelstein et al [41] review, the results revealed that the health status of patients among groups did not differ with the use of communication systems.

##### Satisfaction or Dissatisfaction of Patients of Using ICTs

Patients’ satisfaction with ICTs was documented in five reviews that demonstrated positive effects [41,44,54,55,58], three that showed negative effects [54,55,58], and two that showed no effect [55,58]. Patient results indicated their degree of satisfaction or dissatisfaction with ICTs; their acceptance, acceptability, and receptiveness of their usage of ICTs; and their appreciation for being able to schedule videoconferences about topics of their choice [44,54,58]. The results were presented in terms of usefulness (or uselessness); perceived and actual benefits or advantages, such as accessibility and flexibility [54]; ease of use, usability, complexity; and the degree to which the ICTs were well-designed and functioned fully [41,55,58]. Some patients were confident in using ICTs [44], whereas others were concerned about the confidentiality of their health information [58]. Results from the Husebo and Storm [54] review indicate that patients who had visual contact with nurses through communication systems felt cared for and perceived a sense of connection.

### Summary Description of eHealth Domains Related to Specific Themes

On the basis of the content of Table 3, we propose a summary description of which eHealth domains cover specific themes of nursing care.

#### Management Systems

The only eHealth domain reported to influence the documentation time was management systems, such as electronic nursing documentation [60], CCIS [47], CPOE, eMAR [43], and EHRs [53]. The other themes reported with these systems were time spent on patient care; time management; information quality and access, intra and interprofessional collaboration; quality of documentation; nurses’ competencies and skills; assessment, care planning, and evaluation; nurses’ perspectives of the quality of care provided; empowerment; and satisfaction or dissatisfaction of nurses and patients using ICTs.

#### Communication Systems

Communication systems was the only eHealth domain found to be applicable to the themes of nurse-patient relationship, autonomy for nurses in their role, and patients’ functional status. These themes were also discussed related to communication systems: teaching patients and families, knowledge update and utilization; intra and interprofessional collaboration; quality of documentation; assessment, care planning, and evaluation; communication and care coordination; nurses’ perspectives of the quality of care provided; satisfaction or dissatisfaction of nurses and patients using ICTs; patient comfort and quality of life related to care; and patients’ empowerment.

#### Computerized Decision Support Systems (CDSSs)

CDSSs are mentioned in nurses' practice environment dimension (3/4): knowledge updating and utilization; information quality and access; and intra and interprofessional collaboration. Regarding the nurses’ competencies and skills, CDSSs are involved with decision-making processes. Some other themes discussed in relation to CDSSs are assessment, care planning, and evaluation; teaching of patients and families; nurses’ perspectives of the quality of care provided; satisfaction or dissatisfaction of nurses and patients using ICTs; and patient comfort and quality of life related to care.

## Discussion

### Summary of Main Results

This overview allowed a broad understanding of the dimensions of nursing care influenced by using ICTs for providing care. Regarding the primary outcomes of interest, the themes that were most frequently reported are documentation time; assessment, care planning, and evaluation; nurses’ perspective of the quality of care provided; information quality and access; and time spent for patient care. For secondary outcomes, satisfaction or dissatisfaction of nurses and patients using ICTs was frequently mentioned.

### Discussion of Results With Respect to the First Function of the NCPF

In relation to the first function of the NCPF (*acquiring, deploying, and maintaining nursing resources*), many reviews outlined outcomes linked to “time.” The use of ICTs affected time management, time spent for patient care, and documentation time. This theme could also refer to a dimension of the NCPF called *maintenance and economic sustainability of the nursing staff* [18]. Sustainability refers to the importance of having quality resources at the lowest cost. This dimension highlights productivity and the necessity to optimize the outputs produced from a given set of inputs; in other words, to minimize the amount of nursing tasks, materials, and equipment without sacrificing the quality of nursing services. The “time” dimension can be understood in terms of how ICTs can impact staff, productivity, optimization of the staff’s time management, and resources utilization. We do believe that time is an interesting outcome related to the resources of the overall structure (nursing staff), but it does not reflect directly on how ICTs can transform or support what nurses do (nursing activities or interventions) within their actual scope of practice. Considering our results, we do not believe that further research should focus on “time” in order to better understand the effects of ICTs on nursing care (and specifically, on nursing processes).

This review did not explore other dimensions and indicators related to the first function of the NCPF, such as nursing staff supply. These dimensions include quantity and quality indicators. As an example, it would be interesting to explore whether the availability of ICTs in specific health care settings impacts the quantity of nurses needed to perform nursing services.

Another relevant topic would be to probe whether ICTs act as facilitator or motivator to enhance nurses’ working conditions, or serve as a barrier that inhibits them. To what extent can ICTs create favorable conditions that attract nurses and reinforce stability in the workforce? A systematic review was undertaken on the effect of ICTs on retention and recruitment of health care professionals [62]. The results revealed that, in 9 out of the 13 studies, ICT use demonstrated a positive, though often indirect, influence on recruitment and retention. The influence of ICTs on retention of nurses was also examined in a qualitative study [63]. The results highlighted various impact of ICTs on nurse retention (ie, little or no impact, unclear impact, or indirect positive impact).

### Discussion of Results With Respect to the Second Function of the NCPF

The three dimensions corresponding to the second function, *transforming nursing resources into nursing services*, are nurses’ practice environment, nursing processes, and professional satisfaction. The themes “knowledge updating and utilization” and “communication and care coordination” were not explicitly described in the NCPF and we used them from the instrument of “actual scope of nursing practice” [32]. The indicator “scope of practice” is included in the nursing processes of the NCPF, but there are no explicit underlying subindicators.

The “information quality and access” theme was analyzed as an effect of ICTs on nurses’ practice environment. In other words, ICTs are seen as a potential way to support nursing work by allowing them to get access to various sources of information and clinical data. The theme “quality of documentation” is not part of the nurses’ practice environment because it is linked to what nurses do as activities.

The capacity of nurses to deliver nursing interventions is intimately and consistently linked with organizational processes that capture the nursing practice context and mediate its outcomes [64,65]. These processes, defined as interventions, support nursing work and sustain a professional environment [66]. We hypothesize that, if nurses have access to a comprehensive set of information about patients, this would trickle down on nursing processes, such as quality of documentation, assessment, care planning, and evaluation. It would also impact communication and care coordination to benefit patient outcomes.

A surprising result is the following: only one review mentioned nurse autonomy in relation to the use of ICTs [59]. It would be interesting to know more about questions such as: How can we define “autonomy” in a context in which nurses use or are exposed to ICTs to provide nursing care? How can ICTs support or influence nurse autonomy? Can ICTs be a required training tool in nurses’ practice environments to support their own autonomy?

The NCPF model reflects the deployment of nurses’ full scope of practice, including assessment, planning, and evaluation; problem and symptom management; health promotion and illness prevention; care coordination; and discharge planning, which are conceptualized through interventions and processes in the model.

From a health care provider perspective, these processes grasp the technical elements of care and reflect the extent to which staff are capable of using and mobilizing their competencies to deploy their entire scope of practice. These processes demonstrate the capability of nurses to engage the needs of patients [18]. Our results show that, in reference to the processes described in the NCPF, few such processes have been described in the studies included in this overview. However, assessment, care planning, and evaluation are the most cited themes in the nursing processes dimension, followed by teaching of patients and families and, finally, by communication and care coordination. Despite these outcomes, it would be helpful to conduct primary studies on how ICTs could influence or support other nursing processes, such as problem and symptom management, health promotion and illness prevention, and discharge planning.

Nurses’ professional satisfaction is conceived as the result of nursing processes. Our results revealed two facets of this satisfaction: nurses’ perspective of the quality of care provided and nurses’ satisfaction or dissatisfaction using ICTs. The NCPF included additional indicators that were not mentioned in the included reviews to capture the nurses’ professional satisfaction: having the time to do their job and the enjoyment derived from it.

### Discussion of Results With Respect to the Third Function of the NCPF

We believe that nursing-sensitive outcomes, which are the “patient outcomes,” are underrepresented in our overview because of our inclusion criteria that focused on reviews of the impact or effect of ICTs on nursing resources and services. Thus, patient outcomes were only considered if nursing outcomes were reported. This means that we included patients’ outcomes as primary outcomes as long as they fell within the usage of ICTs by nurses, and then, when outcomes related to the second function of the NCPF (nursing services and processes) were reported. Dubois and colleagues [67] undertook a systematic work including three literature reviews to identify the priority indicators in evaluating the nursing contribution to quality of care. The results revealed that the most frequently examined nursing sensitive outcomes are pressure ulcers, medication administration errors, urinary infections by catheter, and falls. These indicators are located in the “risk outcomes and safety” dimension of the NCPF. Despite this, there are several systematic reviews on the effects of ICTs on patients’ outcomes [68-71]. However, these reviews do not necessarily explore the impact of ICTs on nursing services and processes (second function of the NCPF) when considering patients’ outcomes.

### Strengths and Potential Biases

There are many strengths of this overview. First, it employed a comprehensive search strategy, which was developed and implemented by a medical librarian. Second, data extraction and quality assessment were conducted by three reviewers working independently. Third, the data extraction process was done with the use of the NCPF, which supported the organization and the analysis of results. This framework supported reflection on the way ICTs could influence specific aspects of nursing care. Some new, redefined, or adapted dimensions and indicators have been suggested in the framework: time management, time spent for patient care and documentation time, information quality and access, quality of documentation, knowledge updating and utilization as part of the nurses' practice environment, communication and care coordination, and nurse and patient satisfaction or dissatisfaction regarding their use of ICTs. Fourth, one of the authors of the NCPF (CAD) challenged the analysis and interpretation of the results. Some debriefing meetings were held to discuss the way the themes were presented related to their organization in the NCPF (under specific subsystems, functions, dimensions, and indicators).

There are also limitations to this overview. First, as mentioned by other authors [27,72], we were limited by the information provided by the review authors. The granularity of details available was limited and some information was lacking regarding both the description of ICTs (eg, their features, components, contexts of use, and area of practice) as well as findings regarding the dimensions of nursing care influenced by ICTs. Therefore, it was not possible to make significant conclusions about how a specific ICT influenced one or many indicators (themes) of nursing care, and it was challenging to categorize these extracted findings (impact of ICTs) within the NCPF. A comprehensive description of interventions (ICTs) would have been helpful. Further research could be done to gain knowledge about how a specific ICT used in a certain area of practice can impact on one or many dimensions and indicators of nursing care.

Third, the nature of the topic was not easy to capture in the reported data of systematic reviews. It was difficult to establish if nurses experienced changes in their practice with the use of ICTs, or if instead they believed that ICTs would change their practice and work environment without really experiencing these transformations. Some outcomes related to the use of ICTs are reported in terms of “barriers.” However, it is not always clear if it is a barrier to use ICTs or an effect or impact of having used them. Systematic reviews on the determinants of nurses’ acceptance and use of ICTs are plenty [31,73-75], but do not inform on the real effects of ICTs on nursing practice.

Fourth, we used AMSTAR to assess methodological quality of qualitative and mixed-method reviews even if this tool was not developed for types of reviews other than quantitative using mainly RCT designs. The results of this work should be interpreted with caution. Although it provides a broad perspective on the phenomenon of interest, the main shortcoming of a review of systematic reviews is the heterogeneity in terms of population, interventions (types of ICTs), types of reviews, and the variety of outcomes, which might lead to the possibility of biased conclusions. For further research and methodological development in this domain, we strongly recommend a consolidated tool to evaluate the quality of different types of reviews on a common scale. The results of the assessment of methodological quality of mixed-method and qualitative reviews must be interpreted with caution, considering that AMSTAR is not used and designed for that purpose. In fact, some criteria do not fit the specificities of other types of reviews because there are no gold standards or guidelines allowing us to perform this task. Consequently, mixed-method and qualitative reviews started with a lower score, which cannot lead to a judgment about the likely bias and methodological limitations inherent in the majority of reviews summarized in Table 2.

Finally, this overview draws a picture of the reality of ICTs that covered a period extended from 2002 to the start of 2015. The emerging or novel ICTs that have been published from 2015 until now could not be captured.

### Differences Between Protocol and Overview

As stated in the protocol [25], one of the objectives was to explore whether specific categories of ICTs (management systems, communication systems, CDSSs, or information systems) could have an impact on nursing care. As mentioned earlier, the heterogeneity of reviews and the lack of granularity regarding extracted data or information were some reasons why we could not pursue the initial objective.

When we planned this overview, we were particularly interested in the dimensions of nursing care inherent to the second and the third function of the NCPF, which are nurses’ practice environment, nursing processes, professional satisfaction (second function or subsystem), and nursing-sensitive outcomes (third function or subsystem). Throughout the data extraction process, we realized that some outcomes, particularly those related to the time and efficiency, were frequently mentioned. We then decided to extract these results based on their frequency and their impact on the nursing care.

### Authors’ Conclusions

To the best of our knowledge, this is the first attempt to draw a broad understanding and a schematization of specific dimensions and indicators of nursing care influenced by ICTs. Using the NCPF was useful to illustrate the way ICTs can impact 3 subsystems (nursing resources, nursing services or processes, and nursing sensitive outcomes or patients’ outcomes), 5 dimensions, and 19 themes corresponding to the NCPF indicators. Findings of this overview are a good starting point from which we could deepen our conceptualization on the way nursing care system performance can be affected by ICTs. According to a systemic perspective, it is plausible to believe that the adoption and implementation of ICTs in the nursing care system must be addressed under a multidimensional perspective, considering that the 3 subsystems are interrelated. If nurses use ICTs to support their interventions, and the impact of such ICTs are positive or negative on the work they do, this could possibly reverberate on patient outcomes. We have to keep this broad representation in mind when it will be the time to plan and to implement emerging ICTs in health care settings.

### Takeaway Messages

Using the NCPF was relevant to draw a broad, multidimensional, and a system-based perspective on the dimensions and indicators of nursing care that can be impacted by ICTs.

ICTs have a mixed impact on 19 indicators related to nursing care: documentation time, time spent for patient care, time management, knowledge updating and utilization, information quality and access, nurse autonomy, intra and interprofessional collaboration, nurses competencies-skills, nurse-patient relationship, quality of documentation, assessment, care planning and evaluation, teaching of patients and families, communication and care coordination, nurses’ perspectives of the quality of care provided, patient comfort and quality of life related to care, empowerment, functional status, and satisfaction or dissatisfaction of nurses and patients using ICTs.

Management systems, including, for instance, electronic nursing documentation system, CCIS, CPOE, eMAR, and EHRs, have been discussed exclusively with the theme “documentation time” (in the included reviews).

Communication systems have been described exclusively regarding nurse-patient relationship, autonomy for nurses in their role, and patients’ functional status (eg, physical, cognitive, and psychosocial functional capacity).

## Appendix

Multimedia Appendix 1 Search strategies.

Multimedia Appendix 2 List of included reviews.

Multimedia Appendix 3 Excluded articles and reasons for exclusion.

Multimedia Appendix 4 General characteristics of included reviews.

Multimedia Appendix 5 Review objectives, limitations, and main conclusions.

Multimedia Appendix 6 eHealth domains, interventions, and comparisons.

## Abbreviations

ADE: adverse drug event
ADL: activities of daily living
AMSTAR: assessment of multiple systematic reviews
APN: advanced practice nurse
BCMA: bar-coded medication administration
CCIS: critical care information system
CDSSs: computerized decision support systems
CINAHL: Cumulative Index to Nursing and Allied Health Literature
COPD: chronic obstructive pulmonary disease
CPIS: computerized patient information systems
CPOE: computerized provider order entry
CS: communication systems
D-RHIS: disease-specific regional health care information systems
ECG: electrocardiogram
ED: emergency department
EHR: electronic health record
eMAR: electronic medication administration record systems
EMR: electronic medical record
EPR: electronic personal record
ES-NIS: expert system nursing information system
EWS: early warning score
HCPs: health care providers
HIT: health information technology
ICTs: information and communication technologies
ICU: intensive care unit
I-RHIS: integrated regional health care information systems
IT: information technology
LOS: length of stay
MeSH: Medical subject heading
MMIT: medication management health information technology
MS: management systems
NCPF: nursing care performance framework
NHS: National Health Service
ORA: organization risk analyzer
PCC: patient-centered care
PDA: personal digital assistant
PHR: personal health record
PICOs: participants, interventions, comparisons, outcomes and studies
PRISMA: Preferred Reporting Items for Systematic Reviews and Meta-Analysis
RCT: randomized controlled trial
RHIO: regional health care information organization
RHIS: regional health care information system
RN: registered nurse
SMS: short message service
TDMD: therapeutic drug monitoring and dosing
WAP: wireless application protocol
