# Supplementary material for: Impact of Information and Communication Technologies on Nursing Care: Results of an Overview of Systematic Reviews
Source: J Med Internet Res. 2017 Apr 25;19(4):e122. doi: 10.2196/jmir.6686 (PMC5424122; doi:10.2196/jmir.6686)
Supplement: Multimedia Appendix 1 [file jmir_v19i4e122_app1.pdf]

Appendix 1: Search strategies

PubMed

Search	Query	Results
#57	(#56 and #52)	1,048
#56	(#10 and #55)	9,698
#55	(#11 or #12 or #13 or #14 or #15 or #16 or #19 or #20 or #21 or #22 or #23 or #24 or #25 or #26 or #27 or #28 or #29 or #33 or #34 or #35 or #37 or #38 or #39 or #40 or #41 or #42 or #43 or #44)	103,615
#54	(#10 and #52 and #53)	1,400
#53	(#11 or #12 or #13 or #14 or #15 or #16 or #18 or #19 or #20 or #21 or #22 or #23 or #24 or #25 or #26 or #27 or #28 or #29 or #33 or #34 or #35 or #37 or #38 or #39 or #40 or #41 or #42 or #43 or #44)	178,564
#52	(systematic[sb] OR meta-analysis[pt] OR meta-analysis as topic[mh] OR meta-analysis[mh] OR meta analy*[tw] OR metanaly*[tw] OR metaanaly*[tw] OR met analy*[tw] OR integrative research[tiab] OR integrative review*[tiab] OR integrative overview*[tiab] OR research integration*[tiab] OR research overview*[tiab] OR collaborative review*[tiab] OR collaborative overview*[tiab] OR systematic review*[tiab] OR technology assessment*[tiab] OR technology overview*[tiab] OR "Technology Assessment, Biomedical"[mh] OR HTA[tiab] OR HTAs[tiab] OR comparative efficacy[tiab] OR comparative effectiveness[tiab] OR outcomes research[tiab] OR indirect comparison*[tiab] OR ((indirect treatment[tiab] OR mixed-treatment[tiab]) AND comparison*[tiab]) OR Embase*[tiab] OR Cinahl*[tiab] OR systematic overview*[tiab] OR methodological overview*[tiab] OR methodologic overview*[tiab] OR methodological review*[tiab] OR methodologic review*[tiab] OR quantitative review*[tiab] OR quantitative overview*[tiab] OR quantitative synthes*[tiab] OR pooled analy*[tiab] OR Cochrane[tiab] OR Medline[tiab] OR Pubmed[tiab] OR Medlars[tiab] OR handsearch*[tiab] OR hand search*[tiab] OR meta-regression*[tiab] OR metaregression*[tiab] OR data synthes*[tiab] OR data extraction[tiab] OR data abstraction*[tiab] OR mantel haenszel[tiab] OR peto[tiab] OR der-simonian[tiab] OR dersimonian[tiab] OR fixed effect*[tiab] OR "Cochrane Database Syst Rev"[Journal:__jrid21711] OR "health technology assessment winchester, england"[Journal] OR "Evid Rep Technol Assess (Full Rep)"[Journal] OR "Evid Rep Technol Assess (Summ)"[Journal] OR "Int J Technol Assess Health Care"[Journal] OR "GMS Health Technol Assess"[Journal] OR "Health Technol Assess (Rockv)"[Journal] OR "Health Technol Assess Rep"[Journal])	316,978
#44	(smartphone or smart phone)	1,295
#43	Cellular Phone[mesh]	5,014

#42	Medical Order Entry Systems[mesh]	1,493
#41	Health Records, Personal[mesh]	718
#40	Reminder Systems[mesh]	2,244
#39	Health Information Systems[mesh]	299
#38	Decision Support Systems, Clinical[mesh]	4,838
#37	Electronic Mail[mesh]	1,866
#35	Computers, Handheld[mesh]	2,323
#34	web-based intervention*	401
#33	(web site* or website*)	18,625
#29	(sms or short message service)	5,156
#28	pda*	10,805
#27	personal digital assistant*	898
#26	ict	3,494
#25	("information and communication technolog*")	12,717
#24	information technolog*	13,553
#23	telecare	2,508
#22	(ehealth or e-health)	20,101
#21	telehealthcare	62
#20	telehealth	18,529
#19	Public Health Informatics	3,883
#16	Electronic Health Record*	9,904
#15	Telenursing	210
#14	(Telemedicine or tele medicine or tele-medicine)	18,314
#13	remote communication*	37
#12	Remote Consultation*	3,709
#11	Decision Making, Computer-Assisted/nurs*	925
#10	(#1 or #2 or #3 of #4 or #5 or #6 or #7 or #8 or #9)	732,152
#9	Nurs*	731,812
#8	Nursing [Subheading]	116,730
#7	Nursing Diagnosis [Mesh]	3,841

#6	Nursing Care [Mesh]	118,176
#5	Evidence-Based Nursing [Mesh]	2,184
#4	Advanced Practice Nursing [Mesh]	834
#3	Nursing Assessment[MeSH Major Topic]	12,306
#2	Nurse's Role[MeSH Major Topic]	15,805
#1	Nurse's Practice Patterns[MeSH Major Topic]	783

CINAHL

Search	Query	Results
#43	#41 AND #42	475
#42	(TI (systematic* n3 review*)) or (AB (systematic* n3 review*)) or (TI (systematic* n3 bibliographic*)) or (AB (systematic* n3 bibliographic*)) or (TI (systematic* n3 literature)) or (AB (systematic* n3 literature)) or (TI (comprehensive* n3 literature)) or (AB (comprehensive* n3 literature)) or (TI (comprehensive* n3 bibliographic*)) or (AB (comprehensive* n3 bibliographic*)) or (TI (integrative n3 review)) or (AB (integrative n3 review)) or (JN “Cochrane Database of Systematic Reviews”) or (TI (information n2 synthesis)) or (TI (data n2 synthesis)) or (AB (information n2 synthesis)) or (AB (data n2 synthesis)) or (TI (data n2 extract*)) or (AB (data n2 extract*)) or (TI (medline or pubmed or psyclit or cinahl or (psycinfo not “psycinfo database”) or “web of science” or scopus or embase)) or (AB (medline or pubmed or psyclit or cinahl or (psycinfo not “psycinfo database”) or “web of science” or scopus or embase)) or (MH “Systematic Review”) or (MH “Meta Analysis”) or (TI (meta-analy* or metaanaly*)) or (AB (meta-analy* or metaanaly*))	79,479
#41	(#1 OR #2 OR #3 OR #4 OR #5 OR #6 OR #7 OR #8 OR #9 OR #10) AND (#39 AND #40)	10,745
#40	#1 OR #2 OR #3 OR#4 OR #5 OR #6 OR #7 OR #8 OR #9	96,152
#39	(#11 OR #12 OR #13 OR #14 OR #15 OR #16 OR #17 OR #18 OR #19 OR #20 OR #21 OR #22 OR #23 OR #24 OR #25 OR #26 OR #27 OR #28 OR #29 OR #30 OR #31 OR #32 OR #33 OR #34 OR #35 OR #36 OR #37 OR #38)	299,359
#38	TX smartphone or smart phone	4,676
#37	MH Wireless Communications	9,143
#36	MH Electronic Order Entry	2,262
#35	MH Medical Records, Personal	740

#34	MH Computers, Hand-Held	3,094
#33	MH Reminder Systems	1,743
#32	MH Health Information Systems	1,782
#31	MH Decision Support Systems, Clinical	2,649
#30	MH Electronic Mail	4,453
#29	TX web-based intervention*	7,779
#28	TX (web site* or website*)	132,221
#27	TX (sms or short message service)	9,272
#26	TX pda*	4,038
#25	TX personal digital assistant*	4,637
#24	TX ict	4,674
#23	TX information and communication technolog*	40,595
#22	TX information technolog*	135,116
#21	TX telecare	2,511
#20	TX (ehealth or e-health)	5,190
#19	TX telehealthcare	86
#18	TX telehealth	6,574
#17	TX Public Health Informatics	9,436
#16	TX Electronic Health Record*	39,897
#15	TX Telenursing	1,857
#14	TX (Telemedicine or tele medicine or tele-medicine)	12,775
#13	TX remote communication*	10,314
#12	MH Remote Consultation	1,166
#11	MH Decision Making, Computer Assisted	1,021
#9	MH Nursing Diagnosis	3,726
#8	MH Nursing Care	16,328
#7	MH Nursing Practice, Evidence-Based	7,644
#6	MH Advanced Nursing Practice	7,886
#5	MH nursing assessment	15,029
#4	MH nursing role	41,332

#3	MH practice patterns	9,901
#2	MM "Practice Patterns"	6,282
#1	MJ Practice Patterns	6,377

Cochrane

Search	Query	Results
#21	DARE, Cochrane Reviews, HTA	2,502
#20	#1 and #19	4,332
#19	#2 or #3 or #4 or #5 or #6 or #7 or #8 or #9 or #10 or #11 or #12 or #13 or #14 or #15 or #16 or #17 or #18	20,774
#18	smartphone or smart phone	162
#17	web-based intervention*	1,679
#16	web site* or website*	5,009
#15	sms or short message service	684
# 14	pda*	604
#13	personal digital assistant*	230
#12	ict	230
#11	information and communication technolog*	931
#10	information technolog*	9,143
#9	telecare	290
#8	ehealth or e-health	381
#7	telehealth*	371
#6	Public Health Informatics	93
#5	Electronic Health Record*	7,326
#4	Telenursing	37
#3	Telemedicine or tele medicine or tele-medicine	1,607
#2	remote communication*	302
#1	nurs*	27,166

Embase

Search	Query	Results
#50	#39 and #49	817
#49	#40 or #41 or #42 or #43 or #44 or #45 or #46 or #47 or #48	245,837
#48	comparative near/3 (efficacy or effectiveness)	20,459
#47	meta next/1 regression* or metaregression*	3,768
#46	met next/1 analy* or metanaly* or technology next/1 assessment* or hta or htas or technology next/1 overview* or technology next/1 appraisal*	25,758
#45	'mantel haenszel' or peto or 'der simonian' or dersimonian or fixed next/1 effect* or latin next/1 square*	19,314
#44	handsearch* or (hand and search*)	13,339
#43	data next/1 synthes* or data next/1 extraction* or data next/1 abstraction*	20,456
#42	integrative near/3 (review* or overview*) or collaborative near/3 (review* or overview*) or pool* near/3 analy*	16,729
#41	quantitative near/3 (review* or overview* or synthes*) or research near/3 (integrati* or overview*)	21,348
#40	'systematic review'/exp or 'meta-analysis'/exp	137,848
#39	#10 and #38	27,532
#38	#11 or #12 or #13 or #14 or #15 or #16 or #17 or #18 or #19 or #20 or #21 or #22 or #23 or #24 or #25 or #26 or #27 or #28 or #29 or #30 or #31 or #32 or #33 or #34 or #35 or #36 or #37	299,928
#37	smartphone or smart next/1 phone	1,714
#36	'cellular phone'/exp	8,515
#35	'medical order entry systems'/exp	17,424
#34	'health records, personal'/exp	147,252
#33	'reminder systems'/exp	1,521
#32	‘health information systems'/exp	14,489
#31	'decision support systems, clinical'/exp	13,324
#30	‘electronic mail'/exp	10,032
#29	'computers, handheld'/exp	13,685

#28	'web based' next/1 intervention*	469
#27	website* or web next/1 site*	24,402
#26	sms or 'short message service'	6,528
#25	pda*	19,566
#24	'personal digital' next/1 assistant*	1,348
#23	ict	5,592
#22	communication next/1 technolog*	2,884
#21	information next/1 technolog*	24,938
#20	ehealth*	1,619
#19	telecar*	2,831
#18	telehealth*	3,240
#17	'public health informatics'	289
#16	'electronic health record'	3,421
#15	telenursing	226
#14	telemedicine or 'tele medicine'	17,460
#13	remote next/1 communication*	42
#12	remote next/1 consultation*	240
#11	'decision making, computer-assisted'/exp	13,324
#10	#1 or #2 or #3 or #4 or #5 or #6 or #7 or #8 or #9	824,943
#9	nurs*	824,501
#8	'nursing'/exp	339,695
#7	'nursing diagnosis'/exp	3,662
#6	'nursing care'/exp	30,587
#5	'evidence based nursing'/exp	2,268
#4	'advanced practice nursing'/exp	979
#3	'nursing assessment'/exp	28,476
#2	'nurse attitude'/exp	33,230
#1	'nursing practice'/exp	3,503

Epistemonikos

Query	Results
(nurs* AND tele* OR information technolog* OR communicati* technolog* or ICT or emr or ehr or electronic health record* or electronic medical record*)	894

Web of Science

Search	Query	Results
# 25	#24 AND #21	451
# 24	#23 OR #22	Approximately 651,677
# 23	TOPIC: (systematic review)	Approximately 306,063
# 22	TOPIC: (meta-analysis or metaanalysis or meta analysis)	Approximately 423,202
# 21	#20 AND #1	Approximately 18,415
# 20	#19 OR #18 OR #17 OR #16 OR #15 OR #14 OR #13 OR #12 OR #11 OR #10 OR #9 OR #8 OR #7 OR #6 OR #5 OR #4 OR #3 OR #2	Approximately 635,643
# 19	TOPIC: (smartphone or smart phone)	Approximately 13,177
# 18	TOPIC: (Electronic Mail)	Approximately 10,054
# 17	TOPIC: (web site* or website*)	Approximately 119,442
# 16	TOPIC: (sms or short message service)	Approximately 19,538
# 15	TOPIC: (pda*)	Approximately 47,576
# 14	TOPIC: (personal digital assistant*)	2,732
# 13	TOPIC: (ict)	Approximately 28,076

# 12	TOPIC: (information and communication technolog*)	Approximately 57,260
# 11	TOPIC: (information technolog*)	Approximately 342,198
# 10	TOPIC: (telecare)	977
# 9	TOPIC: (ehealth or e-health)	Approximately 8,021
# 8	TOPIC: (telehealth)	Approximately 6,320
# 7	TOPIC: (telehealth)	Approximately 6,320
# 6	TOPIC: (Public Health Informatics)	Approximately 5,530
# 5	TOPIC: (Electronic Health Record*)	Approximately 36,111
# 4	TOPIC: (Telenursing)	252
# 3	TOPIC: (Telemedicine or tele medicine or tele-medicine)	Approximately 33,391
# 2	TOPIC: (remote communication*)	Approximately 20,406
# 1	TOPIC: (nurs*)	Approximately 1,020,251
