# Supplementary material for: Impact of Information and Communication Technologies on Nursing Care: Results of an Overview of Systematic Reviews
Source: J Med Internet Res. 2017 Apr 25;19(4):e122. doi: 10.2196/jmir.6686 (PMC5424122; doi:10.2196/jmir.6686)
Supplement: Multimedia Appendix 2 [file jmir_v19i4e122_app2.pdf]

Appendix 2 : List of included reviews

Anderson JA, Willson P. Clinical decision support systems in nursing: synthesis of the science for evidence-based practice. Comput Inform Nurs. 2008;26(3):151-158. PMID: 2009922759

Bartoli L, Zanaboni P, Masella C, Ursini N. Systematic review of telemedicine services for patients affected by chronic obstructive pulmonary disease (COPD). Telemed J E Health. 2009;15(9):877-883. PMID: 19919194

Bowles KH, Baugh AC. Applying research evidence to optimize telehomecare. J Cardiovasc Nurs. 2007;22(1):5-15. PMID: 17224692

Carrington JM, Tiase VL. Nursing informatics year in review. Nurs Adm Q. 2013;37(2):136-143. PMID: 23454993

Dowding D, Randell R, Gardner P, et al. Dashboards for improving patient care: Review of the literature. Int J Med Inform. 2015 Oct 12;84(2):87-100. PMID: 25453274

Finkelstein J, Knight A, Marinopoulos S, et al. Enabling patient-centered care through health information technology. Rockville, MD: Agency for Healthcare Research and Quality, 2012 Jun. Report No.: 1530-4396. PMID: 24422882

Free C, Phillips G, Watson L, et al. The effectiveness of mobile-health technologies to improve health care service delivery processes: a systematic review and meta-analysis. PLoS Med. 2013;10(1):e1001363. PMID: 23458994

Georgiou A, Prgomet M, Paoloni R, et al. The effect of computerized provider order entry systems on clinical care and work processes in emergency departments: a systematic review of the quantitative literature. Ann Emerg Med. 2013 Jun;61(6):644-653. PMID: 23548404

Husebo AML, Storm M. Virtual visits in home health care for older adults. ScientificWorldJournal. 2014;689873. PMID: 25506616

Jones JF, Brennan PF. Telehealth interventions to improve clinical nursing of elders. Annu Rev Nurs Res. 2002;20:293-322. PMID: 12092513

Kelley TF, Brandon DH, Docherty SL. Electronic nursing documentation as a strategy to improve quality of patient care. J Nurs Scholarsh. 2011 Jun;43(2):154-162. PMID: 21605319

Mador RL, Shaw NT. The impact of a Critical Care Information System (CCIS) on time spent charting and in direct patient care by staff in the ICU: A review of the literature. Int J Med Inform. 2009;78(7):435-445. PMID: 19261544

Maenpaa T, Suominen T, Asikainen P, Maass M, Rostila I. The outcomes of regional healthcare information systems in health care: A review of the research literature. Int J Med Inform. 2009;78(11):757-771. PMID: 19656719

McKibbon KA, Lokker C, Handler SM, et al. Enabling medication management through health information technology (Health IT). Evidence report/technology assessment: 2011 Apr. Report No.: 1530-4396 PMID: 23126642

Meißner A, Schnepp W. Staff experiences within the implementation of computer-based nursing records in residential aged care facilities: a systematic review and synthesis of qualitative research. BMC Med Inform Decis Mak. 2014;14:54. PMID: 24947420

Mickan S, Atherton H, Roberts NW, Heneghan C, Tilson JK. Use of handheld computers in clinical practice: a systematic review. BMC Med Inform Decis Mak. 2014;14:56. PMID: 24998515

Nguyen L, Bellucci E, Nguyen LT. Electronic health records implementation: An evaluation of information system impact and contingency factors. Int J Med Inform. 2014 Nov;83(11):779-796. PMID: 25085286

Nieuwlaat R, Connolly SJ, Mackay JA, et al. Computerized clinical decision support systems for therapeutic drug monitoring and dosing: a decision-maker-researcher partnership systematic review. Implement Sci. 2011;6:90. PMID: 21824384

Poissant L, Pereira J, Tamblyn R, Kawasumi Y. The impact of electronic health records on time efficiency of physicians and nurses: a systematic review. J Am Med Inform Assoc. 2005 Sep-Oct;12(5):505-516. PMID: 15905487

Randell R, Mitchell N, Dowding D, Cullum N, Thompson C. Effects of computerized decision support systems on nursing performance and patient outcomes: a systematic review. J Health Serv Res Policy. 2007 Oct;12(4):242-249. PMID: 17925077

Stevenson JE, Nilsson GC, Petersson GI, Johansson PE. Nurses'™ experience of using electronic patient records in everyday practice in acute/inpatient ward settings: A literature review. Health Informatics J. 2010;16(1):63-72. PMID: 20413414

Urquhart C, Currell R, Grant MJ, Hardiker NR. Nursing record systems: effects on nursing practice and healthcare outcomes. Cochrane Database Syst Rev. 2009;Art. No.: CD002099(Issue 1). PMID: 19160206
