# Supplementary material for: Impact of Information and Communication Technologies on Nursing Care: Results of an Overview of Systematic Reviews
Source: J Med Internet Res. 2017 Apr 25;19(4):e122. doi: 10.2196/jmir.6686 (PMC5424122; doi:10.2196/jmir.6686)
Supplement: Multimedia Appendix 3 [file jmir_v19i4e122_app3.pdf]

Appendix 3. Excluded articles

No outcomes related to nursing practice (no primary findings)	Nurses-related findings inseparable from other healthcare professionals	Had an updated review	Proceeding abstract only (no full review found)	Protocol only (no full review found)	No study in the review	Insufficient details on methodology (doubt on systematization)	Review objective carries on acceptance, attitude and factors of adoption of ICT
Akesson 2007	Arditi 2012	Currell 2003	Clark 2010	Koivunen 2014	Martin 2008	Bakken 2006	Brewster 2013
Anderson 2012	Bassi 2010		MacLure 2011			Merksouris 1995	Gagnon 2012
Atherton 2012	Dorr 2007		MacLure 2014a				Huryk 2010
Chipps 2012	Jennett 2003						McGinn 2011
Clark 2013	Kawamoto 2005						Young 2011
Currell 2000	Keane 2009						
Du 2013	Kukreti 2014						
Durieux 2008	Lindberg 2013						
Gagnon 2010	MacLure 2014b						
Hemens 2011	McGowan 2009						
Holroyd-Leduc 2011	McKibbon 2012						
Inglis 2010	Pearson 2009						
Johansson 2010	Vedel 2013						
Lluch 2011							
Nguyen 2015							
Osborn 2010							
Pappas 2012							
Roine 2001							
Roshanov 2011							
Sant'Anna Vargas 2013							
Sawmynaden 2012							
Shojania Kaveh 2009							
Wei 2011							
Yu 2012							

References of excluded articles

Akesson, K. M., Saveman, B. I., & Nilsson, G. (2007). Health care consumers' experiences of information communication technology-A summary of literature. *International Journal of Medical Informatics, 76*(9), 633-645.

Anderson, L. S., & Enge, K. J. (2012). Education and Information for Practicing School Nurses: Which Technology-Supported Resources Meet Their Needs? *Journal of School Nursing (Sage Publications Inc.), 28*(5), 358-369. doi:10.1177/1059840512443261.

Arditi, C., Rège-Walther, M., Wyatt Jeremy, C., Durieux, P., & Burnand, B. (2012). Computer-generated reminders delivered on paper to healthcare professionals; effects on professional practice and health care outcomes. *Cochrane Database of Systematic Reviews,* (12). Retrieved from http://onlinelibrary.wiley.com/doi/10.1002/14651858.CD001175.pub3/abstract doi:10.1002/14651858.CD001175.pub3

Atherton, H., Sawmynaden, P., Meyer, B., & Car, J. (2012). Email for the coordination of healthcare appointments and attendance reminders. *Cochrane Database of Systematic Reviews,* (8). Retrieved from http://onlinelibrary.wiley.com/doi/10.1002/14651858.CD007981.pub2/abstract doi:10.1002/14651858.CD007981.pub2

Bakken, S. (2006). Informatics for patient safety: a nursing research perspective. *Annu Rev Nurs Res, 24*, 219-254.

Bassi, J., Lau, F., & Bardal, S. (2010). Use of information technology in medication reconciliation: a scoping review. *Ann Pharmacother*, *44*(5), 885-897. doi:10.1345/aph.1M699

Brewster, L., Mountain, G., Wessels, B., Kelly, C., & Hawley, M. (2013). Factors affecting front line staff acceptance of telehealth technologies: a mixed-method systematic review. *J Adv Nurs*. doi:10.1111/jan.12196

Chipps, J., Brysiewicz, P., & Mars, M. (2012). Effectiveness and feasibility of telepsychiatry in resource constrained environments? A systematic review of the evidence. *African Journal of Psychiatry (South Africa), 15*(4), 235-243.

Clarke, M. A., Belden, J. L., Koopman, R. J., Steege, L. M., Moore, J. L., Canfield, S. M., & Kim, M. S. (2013). Information needs and information-seeking behaviour analysis of primary care physicians and nurses: a literature review. Health Info Libr J, 30(3), 178-190. doi:10.1111/hir.12036

Clark, R. A., Inglis, S. C., McAlister, F. A., Ball, J., Lewinter, C., Cullington, D., . . . Cleland, J. G. F. (2010). Results from a systematic review and meta-analysis of remote (non-invasive) monitoring in 8,323 heart failure patients on length of stay, quality of life, knowledge, compliance and satisfaction. *European Journal of Heart Failure, Supplement, 9*, S51-S52.

Currell, R., & Urquhart, C. (2003). Nursing record systems: effects on nursing practice and health care outcomes. *Cochrane Database Syst Rev*(3), Cd002099. doi:10.1002/14651858.cd002099

Currell, R., Urquhart, C., Wainwright, P., & Lewis, R. (2000). Telemedicine versus face to face patient care: effects on professional practice and health care outcomes. *Cochrane Database of Systematic Reviews*(2). doi:10.1002/14651858.CD002098

Dorr, D., Bonner, L. M., Cohen, A. N., Shoai, R. S., Perrin, R., Chaney, E., & Young, A. S. (2007). Informatics systems to promote improved care for chronic illness: a literature review. *J Am Med Inform Assoc, 14*(2), 156-163. doi:10.1197/jamia.M2255

Du, S., Liu, Z., Liu, S., Yin, H., Xu, G., Zhang, H., & Wang, A. (2013). Web-based distance learning for nurse education: a systematic review. *International Nursing Review, 60*(2), 167-177.

Durieux, P., Trinquart, L., Colombet, I., Nies, J., Walton, R., Rajeswaran, A., . . . Burnand, B. (2008). Computerized advice on drug dosage to improve prescribing practice. *Cochrane Database Syst Rev*(3), Cd002894. doi:10.1002/14651858.CD002894.pub2

Gagnon, M. P., Desmartis, M., Labrecque, M., Car, J., Pagliari, C., Pluye, P., . . . Legare, F. (2012). Systematic review of factors influencing the adoption of information and communication technologies by healthcare professionals. *Journal of Medical Systems, 36*(1), 241-277.

Gagnon, M. P., Pluye, P., Desmartis, M., Car, J., Pagliari, C., Labrecque, M., . . . Legare, F. (2010). A systematic review of interventions promoting clinical information retrieval technology (CIRT) adoption by healthcare professionals. *International Journal of Medical Informatics, 79*(10), 669-680.

Hemens, B. J., Holbrook, A., Tonkin, M., Mackay, J. A., Weise-Kelly, L., Navarro, T., . . . Haynes, R. B. (2011). Computerized clinical decision support systems for drug prescribing and management: a decision-maker-researcher partnership systematic review. *Implementation Science, 6*, 89-89.

Holroyd-Leduc, J. M., Lorenzetti, D., Straus, S. E., Sykes, L., & Quan, H. (2011). The impact of the electronic medical record on structure, process, and outcomes within primary care: A systematic review of the evidence. *Journal of the American Medical Informatics Association, 18*(6), 732-737.

Huryk, L. A. (2010). Factors influencing nurses' attitudes towards healthcare information technology. *J Nurs Manag, 18*(5), 606-612. doi:10.1111/j.1365-2834.2010.01084.x

Inglis, S. C., Clark, R. A., McAlister, F. A., Stewart, S., & Cleland, J. G. (2011). Which components of heart failure programmes are effective? A systematic review and meta-analysis of the outcomes of structured telephone support or telemonitoring as the primary component of chronic heart failure management in 8323 patients: Abridged Cochrane Review. *Eur J Heart Fail, 13*(9), 1028-1040. doi:10.1093/eurjhf/hfr039

Jennett, P. A., Hall, L. A., Hailey, D., Ohinmaa, A., Anderson, C., Thomas, R., . . . Scott, R. E. (2003). The socio-economic impact of telehealth: a systematic review. *Journal of Telemedicine and Telecare, 9*(6), 311-320.

Johansson, T., & Wild, C. (2010). Telemedicine in acute stroke management: systematic review (Structured abstract). *International Journal of Technology Assessment in Health Care, 26*(2), 149-155. Retrieved from http://onlinelibrary.wiley.com/o/cochrane/cldare/articles/DARE-12010004058/frame.html

Kawamoto, K., Houlihan, C. A., Balas, E. A., & Lobach, D. F. (2005). Improving clinical practice using clinical decision support systems: a systematic review of trials to identify features critical to success (Structured abstract). *Bmj, 330*(4), 765. Retrieved from http://onlinelibrary.wiley.com/o/cochrane/cldare/articles/DARE-12005008198/frame.html

Keane, M. G. (2009). A review of the role of telemedicine in the accident and emergency department. *Journal of Telemedicine and Telecare, 15*(3), 132-134. doi:10.1258/jtt.2009.003008

Koivunen, M., & Saranto, K. (2014). Nursing professionals' experiences of the facilitators and barriers to the use of telehealth applications: - A systematic review of qualitative evidence. *JBI Database of Systematic Reviews and Implementation Reports, 10*(57), 3894-3906.

Kukreti, V., Cosby, R., Cheung, A., & Lankshear, S. (2014). Computerized prescriber order entry in the outpatient oncology setting: From evidence to meaningful use. *Current Oncology, 21*(4), e604-e612.

Lindberg, B., Nilsson, C., Zotterman, D., Soderberg, S., & Skar, L. (2013). Using Information and Communication Technology in Home Care for Communication between Patients, Family Members, and Healthcare Professionals: A Systematic Review. *Int J Telemed Appl, 2013*, 461829. doi:10.1155/2013/461829

Lluch, M. (2011). Healthcare professionals' organisational barriers to health information technologies-A literature review. *International Journal of Medical Informatics, 80*(12), 849-862.

MacLure, K., Stewart, D., McHattie, L., & Strath, A. (2011). A systematic review of the impact of technology on shared care: Why not ask your pharmacist? *International Journal of Pharmacy Practice, 19*, 98-99.

MacLure, K., Stewart, D., & Strath, A. (2014). Mind the gap: Multidisciplinary team perceptions of ehealth in relation to integrated care. *International Journal of Pharmacy Practice, 22*, 16.

Maclure, K., Stewart, D., & Strath, A. (2014). A systematic review of medical and non-medical practitioners' views of the impact of ehealth on shared care. *European Journal of Hospital Pharmacy: Science and Practice, 21*(1), 54-62.

Martin, S., Kelly, G., Kernohan, W. G., McCreight, B., & Nugent, C. (2008). Smart home technologies for health and social care support. *Cochrane Database of Systematic Reviews*, (4). Retrieved from http://onlinelibrary.wiley.com/doi/10.1002/14651858.CD006412.pub2/abstract doi:10.1002/14651858.CD006412.pub2

McGinn, C. A., Grenier, S., Duplantie, J., Shaw, N., Sicotte, C., Mathieu, L., . . . Gagnon, M. (2011). Comparison of user groups' perspectives of barriers and facilitators to implementing electronic health records: A systematic review. *BMC Medicine, 9*.

McGowan, J., Grad, R., Pluye, P., Hannes, K., Deane, K., Labrecque, M., . . . Tugwell, P. (2009). Electronic retrieval of health information by healthcare providers to improve practice and patient care. *Cochrane Database of Systematic Reviews*, (3). Retrieved from http://onlinelibrary.wiley.com/doi/10.1002/14651858.CD004749.pub2/abstract doi:10.1002/14651858.CD004749.pub2

McKibbon, K. A., Lokker, C., Handler, S. M., Dolovich, L. R., Holbrook, A. M., O'Reilly, D., . . . Roshanov, P. S. (2012). The effectiveness of integrated health information technologies across the phases of medication management: a systematic review of randomized controlled trials. *J Am Med Inform Assoc, 19*(1), 22-30. doi:10.1136/amiajnl-2011-000304

Merkouris, A. V. (1995). Computer-based documentation and bedside terminals. *J Nurs Manag, 3*(2), 81-85.

Nguyen, C., McElroy, L. M., Abecassis, M. M., Holl, J. L., & Ladner, D. P. (2015). The use of technology for urgent clinician to clinician communications: A systematic review of the literature. *International Journal of Medical Informatics, 84*(2), 101-110.

Osborn, C. Y., Mayberry, L. S., Mulvaney, S. A., & Hess, R. (2010). Patient web portals to improve diabetes outcomes: A systematic review. *Current Diabetes Reports, 10*(6), 422-435.

Pappas, Y., Atherton, H., Sawmynaden, P., & Car, J. (2012). Email for clinical communication between healthcare professionals. *Cochrane Database of Systematic Reviews,* (9). Retrieved from http://onlinelibrary.wiley.com/doi/10.1002/14651858.CD007979.pub2/abstract doi:10.1002/14651858.CD007979.pub2

Pearson, S. A., Moxey, A., Robertson, J., Hains, I., Williamson, M., Reeve, J., & Newby, D. (2009). Do computerised clinical decision support systems for prescribing change practice? A systematic review of the literature (1990-2007). *BMC Health Services Research, 9,* 154-154. doi:10.1186/1472-6963-9-154

Roine, R., Ohinmaa, A., & Hailey, D. (2001). Assessing telemedicine: a systematic review of the literature. *Canadian Medical Association Journal, 165*(6), 765-771.

Roshanov, P. S., You, J. J., Dhaliwal, J., Koff, D., Mackay, J. A., Weise-Kelly, L., . . . Haynes, R. B. (2011). Can computerized clinical decision support systems improve practitioners' diagnostic test ordering behavior? A decision-maker-researcher partnership systematic review. *Implementation Science, 6,* 88-88.

Sant'Anna Vargas, G., Lima da Silva, J. L., Marinho Chrizostimo, M., & Pereira Rodrigues, D. (2013). Electronic medical record as a facilitator tool in control of infections related to assistance: integrative review. *Journal of Nursing UFPE, 7*(7S), 4831-4840. doi:10.5205/reuol.4700-39563-1-ED.0707esp201303

Sawmynaden, P., Atherton, H., Majeed, A., & Car, J. (2012). Email for the provision of information on disease prevention and health promotion. *Cochrane Database of Systematic Reviews,* (11). Retrieved from http://onlinelibrary.wiley.com/doi/10.1002/14651858.CD007982.pub2/abstract doi:10.1002/14651858.CD007982.pub2

Shojania, K. G., Ranji, S. R., McDonald, K. M., Grimshaw, J. M., Sundaram, V., Rushakoff, R. J., & Owens, D. K. (2006). Effects of quality improvement strategies for type 2 diabetes on glycemic control: a meta-regression analysis. *Jama, 296*(4), 427-440. doi:10.1001/jama.296.4.427

Vedel, I., Akhlaghpour, S., Vaghefi, I., Bergman, H., & Lapointe, L. (2013). Health information technologies in geriatrics and gerontology: a mixed systematic review. *Journal of the American Medical Informatics Association, 20*(6), 1109-1119.

Wei, I., Pappas, Y., Car, J., Sheikh, A., & Majeed, A. (2011). Computer-assisted versus oral-and-written dietary history taking for diabetes mellitus. *Cochrane Database of Systematic Reviews,* (12). Retrieved from http://onlinelibrary.wiley.com/doi/10.1002/14651858.CD008488.pub2/abstract doi:10.1002/14651858.CD008488.pub2

Young, L. B., Chan, P. S., & Cram, P. (2011). Staff acceptance of tele-ICU coverage: a systematic review. *Chest, 139*(2), 279-288. doi:10.1378/chest.10-1795

Yu, C. H., Bahniwal, R., Laupacis, A., Leung, E., Orr, M. S., & Straus, S. E. (2012). Systematic review and evaluation of web-accessible tools for management of diabetes and related cardiovascular risk factors by patients and healthcare providers (Structured abstract). *Journal of the American Medical Informatics Association, 19*(4), 514-522. Retrieved from http://onlinelibrary.wiley.com/o/cochrane/cldare/articles/DARE-12012041930/frame.html
