# Supplementary material for: Impact of Information and Communication Technologies on Nursing Care: Results of an Overview of Systematic Reviews
Source: J Med Internet Res. 2017 Apr 25;19(4):e122. doi: 10.2196/jmir.6686 (PMC5424122; doi:10.2196/jmir.6686)
Supplement: Multimedia Appendix 4 [file jmir_v19i4e122_app4.pdf]

Appendix 4. General characteristics of included reviews.

Review	Type of review	Design of included studies	Number of studies	Search dates	Target populations (n)	Health care settings
Anderson (2008)	Mixed	Various, including qualitative, experimental, quasi-experimental, and nonexperimental	17	Not reported	Nurses	Emergency department (ED), oncology nursing, in-hospital clinic, long-term care, neonatal unit, critical care
Bartoli (2009)	Mixed	Various including quantitative and qualitative (no specific designs were mentioned)	17 from 40 full-text articles	1996-2008	Patients (1236); nurses (23)	Not specified
Bowles (2007)	Mixed	Various, including prospective, pre- and posttest, group case control, observational, descriptive, randomized trial, intervention group only, pilot study, longitudinal, quasi-experimental, retrospective chart review	19	1995-2005	*For those numbered:* patients (1554), nurses (26)	Home care

Carrington (2013)	Rapid review (mixed)	Various, including qualitative, quantitative, and mixed-methods	80	August 1, 2011 to August 1, 2012	Nurses and nurse managers	Hospital; community; online; education, ambulatory; home health; long-term care
Dowding (2015)	Rapid review (quantitative)	Various, including before-after study, usability study, cluster randomized controlled trial (RCT), noncomparative study, time series analysis	11	November 1996 to December 2012	Patients (20), nurses (not numbered), clinical staff (175), mental health professionals (21), primary care physicians (72), clinicians (258), radiologists (47), anesthesists (29), pharmacists (51)	Primary care practices
Finkelstein (2012)	Mixed	Various, including quantitative (eg, RCT), qualitative, and usability studies	327	Unspecified to July 31, 2010	Physicians, patients, nurses, providers (clinic staff, clinicians), pharmacists	Home health care; clinic; primary care health center; hospital

Free (2013)	Quantitative	Controlled trials	42	January 1990 to September 2010	*For those numbered:* medical students (361), residents (356), physicians (80), second-year baccalaureate nursing students (36), nurse anesthesists (4), undergraduate nurses (87), nursing coordinators (16), nurses (71), patients (3767), patient scans (42), volunteers (62)	Acute care hospital; clinic
Georgiou (2013)	Quantitative	Various including: pre-post design, prospective studies, RCT, time series studies	22	January 1990 to May 2011	*For those numbered:* patients (32182); physicians (126); nurses (26)	ED

Husebo (2014)	An integrative review (mixed)	Various including RCT, descriptive, qualitative, longitudinal study, quasi-experimental with qualitative data, cross-sectional study	12	January 2003 to April 2013	Elderly patients aged 65 years and over (548), nurses (57), professionals caregivers (4), family users (8)	Home health care
Jones (2002)	Integrated review	Various including retrospective review, randomized prospective trial, cross-over design, pair wise comparison, randomized field experience, qualitative	18	1966-2001	Nurses, adult clients of a medical ambulatory clinic (152), patients (188), elderly (68, home care patients), residents long-term facility (120), patients receiving telenursing (22), caregivers of Alzheimer (102)	Medical ambulatory clinic, home care, long-term care facility, psychiatry service
Kelley (2011)	Integrative	Various, including descriptive and cross-sectional designs	24	Articles published over the past 30 years	Nurses	Hospital settings

Mador (2009)	Quantitative	Various including pre-and posttest, prospective observational analysis, RCT, comparative observational design, meta-analysis	12	1937-2008	Nurses, physicians, ward secretaries, respiratory therapists, and technologist	Intensive care unit (ICU)
Maenpaa (2009)	Mixed	Various, including survey research, case study, evaluation or constructive evaluation studies, and multimethodical triangulation	24	1966-2008	*For those numbered:* participants (262), health professionals (318), patients (324), nurses (41), physicians (1390), potential users (250), administrators (8), managers (12), representative of federally qualified clinic (1), local public health officials (2).	Community health centers, health departments and hospitals, provider organizations, academic health centers, and community health information exchange organizations.

McKibbon (2011)	Mixed	Various, including RCT, cohort, case-control, observational, and qualitative studies	789	Fall 2009 to Summer 2010.	Physicians, pharmacists, nurses, junior students, residents, respiratory technicians, health care providers, clinicians, physician assistants, midwives, staff, patients, hospital administrators	Ambulatory care; hospital; ambulatory clinic; acute care; American pediatric hospital; long-term care; elderly home care
Meißner (2014)	Meta-ethnography	Qualitative	7	January 2000 to January 2013	Administrators, director of nursing, nurses, charge nurses, personal carers	Residential aged care facilities
Mickan (2014)	Quantitative	RCT	7	2001-2013	physicians and residents, nursing students, RN[a], patients	Clinic, ED, med-surg wards
Nguyen (2014)	Mixed	Various including single and multiple case studies, survey, ethnography, focus group, secondary	98	2001-2011	Clinicians (34, doctors and nurses), doctors only	Tertiary care, secondary care, primary care, long-

		data analysis, mixed-methods, longitudinal			(27), doctors and other nonclinical participants (4), nurses and nonclinical participants (12), management and/or administration only (8), patients only (5), organizations (4), IT[b] providers and staff	term care facilities
Nieuwlaat (2011)	Quantitative	RCT	33	In previous review: until September 2004; current review: extended search until Jan 2010	Patients (24,627), physicians, advanced practice nurses (APN), physician assistants, pharmacists, other health professionals	Academic centers, hospital outpatients or inpatients, primary care, community-based and subspecialty clinic
Poissant (2005)	Quantitative	Various, including RCT, posttest control studies,	23	1990-2004	Physicians, nurses (215)	Home, hospital, clinic

		one-group pre- and posttest designs.				
Randell (2007)	Quantitative	RCT	9	Up to April 2005. The search was rerun in May 2006.	Nurses (100 and more), patients (more than 24,000)	Primary care, National Health Service (NHS), ICU
Stevenson (2010)	Mixed	Various, including descriptive, cross-sectional, pre- and postquasi-experimental, qualitative	5	2000-2009	Midwives, nurses, nursing assistants, project managers (326)	Acute or inpatient care settings
Urquhart (2009)	Quantitative (Cochrane)	8 RCT; 1 controlled before and after study	9	Update of 2000 and 2003 review; searched up to the end of 2007	Patients and nurses (1846 in total)	Nursing wards, long-term care facility, psychiatric department, ICU

[a]RN: registered nurse.

[b]IT: information technology.

References

Anderson JA, Willson P. Clinical decision support systems in nursing: synthesis of the science for evidence-based practice. Comput Inform Nurs. 2008;26(3):151-158. PMID: 2009922759

Bartoli L, Zanaboni P, Masella C, Ursini N. Systematic review of telemedicine services for patients affected by chronic obstructive pulmonary disease (COPD). Telemed J E Health. 2009;15(9):877-883. PMID: 19919194

Bowles KH, Baugh AC. Applying research evidence to optimize telehomecare. J Cardiovasc Nurs. 2007 Jan-Feb;22(1):5-15. PMID: 17224692

Carrington JM, Tiase VL. Nursing informatics year in review. Nurs Adm Q. 2013;37(2):136-143. PMID: 23454993

Dowding D, Randell R, Gardner P, et al. Dashboards for improving patient care: Review of the literature. Int J Med Inform. 2015 Oct 12;84(2):87-100. PMID: 25453274

Finkelstein J, Knight A, Marinopoulos S, et al. Enabling patient-centered care through health information technology. Rockville, MD: Agency for Healthcare Research and Quality, 2012 Jun. Report No.: 1530-4396 Contract No.: 206. Accessed 206. PMID: 24422882

Free C, Phillips G, Watson L, et al. The effectiveness of mobile-health technologies to improve health care service delivery processes: a systematic review and meta-analysis. PLoS Med. 2013;10(1):e1001363. PMID: 23458994

Georgiou A, Prgomet M, Paoloni R, et al. The effect of computerized provider order entry systems on clinical care and work processes in emergency departments: a systematic review of the quantitative literature. Ann Emerg Med. 2013 Jun;61(6):644-653. PMID: 23548404

Husebo AML, Storm M. Virtual visits in home health care for older adults. ScientificWorldJournal. 2014;Article ID 689873. PMID: 25506616

Jones JF, Brennan PF. Telehealth interventions to improve clinical nursing of elders. Annu Rev Nurs Res. 2002;20:293-322. PMID: 12092513

Kelley TF, Brandon DH, Docherty SL. Electronic nursing documentation as a strategy to improve quality of patient care. J Nurs Scholarsh. 2011 Jun;43(2):154-162. PMID: 21605319

Mador RL, Shaw NT. The impact of a Critical Care Information System (CCIS) on time spent charting and in direct patient care by staff in the ICU: A review of the literature. Int J Med Inform. 2009;78(7):435-445. PMID: 19261544

Maenpaa T, Suominen T, Asikainen P, Maass M, Rostila I. The outcomes of regional healthcare information systems in health care: A review of the research literature. Int J Med Inform. 2009;78(11):757-771. PMID: 19656719

McKibbon KA, Lokker C, Handler SM, et al. Enabling medication management through health information technology (Health IT). Evidence report/technology assessment: 2011 Apr. Report No.: 1530-4396 Contract No.: 201. Accessed 201. PMID: 23126642

Meißner A, Schnepp W. Staff experiences within the implementation of computer-based nursing records in residential aged care facilities: a systematic review and synthesis of qualitative research. BMC Med Inform Decis Mak. 2014;14:54. PMID: 24947420

Mickan S, Atherton H, Roberts NW, Heneghan C, Tilson JK. Use of handheld computers in clinical practice: a systematic review. BMC Med Inform Decis Mak. 2014;14:56. PMID: 24998515

Nguyen C, McElroy LM, Abecassis MM, Holl JL, Ladner DP. The use of technology for urgent clinician to clinician communications: A systematic review of the literature. Int J Med Inform. 2015 Feb;84(2):101-110. PMID: 25444594

Nieuwlaat R, Connolly SJ, Mackay JA, et al. Computerized clinical decision support systems for therapeutic drug monitoring and dosing: a decision-maker-researcher partnership systematic review. Implement Sci. 2011;6:90. PMID: 21824384

Poissant L, Pereira J, Tamblyn R, Kawasumi Y. The impact of electronic health records on time efficiency of physicians and nurses: a systematic review. J Am Med Inform Assoc. 2005 Sep-Oct;12(5):505-516. PMID: 15905487

Randell R, Mitchell N, Dowding D, Cullum N, Thompson C. Effects of computerized decision support systems on nursing performance and patient outcomes: a systematic review. J Health Serv Res Policy. 2007 Oct;12(4):242-249. PMID: 17925077

Stevenson JE, Nilsson GC, Petersson GI, Johansson PE. Nurses'™ experience of using electronic patient records in everyday practice in acute/inpatient ward settings: A literature review. Health Informatics J. 2010;16(1):63-72. PMID: 20413414

Urquhart C, Currell R, Grant MJ, Hardiker NR. Nursing record systems: effects on nursing practice and healthcare outcomes. Cochrane Database Syst Rev. 2009;Art. No.: CD002099(Issue 1). PMID: 19160206
