# Supplementary material for: Impact of Information and Communication Technologies on Nursing Care: Results of an Overview of Systematic Reviews
Source: J Med Internet Res. 2017 Apr 25;19(4):e122. doi: 10.2196/jmir.6686 (PMC5424122; doi:10.2196/jmir.6686)
Supplement: Multimedia Appendix 5 [file jmir_v19i4e122_app5.pdf]

Appendix 5. Review objectives, limitations, and main conclusions.

Review	Review objectives	Review limitations stated by authors	Review authors' conclusions
Anderson (2008)	To present a synthesis of the research literature on the state of nursing science regarding the development, use, and application of clinical decision support systems for the implementation of evidence-based practice in nursing.	None stated	There is a significant knowledge gap on nurse use of CDSSs[a]. Additional theoretical models for the development and testing of CDSSs in nursing are needed to inform and guide nurses on prevention, patient education, and self-management interventions.
Bartoli (2009)	To identify telemedicine services for patients affected by chronic obstructive pulmonary disease (COPD): (1) which telemedicine applications and related organizational models have been adopted for these patients; and (2) the impact of these applications.	None stated	Telemedicine assumes and entails significant changes in work processes. Its adoption resulted in the reconfiguration of existing practices and sociomaterial relationships.

Bowles (2007)	To present a summary and critique of the published empirical evidence about the effects of telehomecare on older adult patients with chronic illness.	None stated	Homecare using communication and monitoring technology is feasible and acceptable to patients and providers and seems to improve outcomes among patients with chronic illness.
Carrington (2013)	To present the findings of a nursing informatics literature review, highlight those publications seen as most influential in the last year and identify common topics and emerging themes in nursing informatics published research.	This review was for a limited period of time. By limiting the literature search to "nursing informatics" and "informatics" as umbrella terms, there is a strong likelihood that additional research articles were not included.	Design, implementation, and evaluation of nursing technologies show promise in nursing efficiencies that may impact patient safety and health care cost.
Dowding (2015)	To provide a comprehensive overview of the current state of evidence for the use of clinical and quality dashboards in health care environments.	Gray literature and studies in languages other than English were not considered. Process of assessing papers was accelerated. Search strategy lacked specificity.	Implementing clinical and/or quality dashboards which provide constant access to information can improve adherence to quality guidelines and may help improve patient outcomes.

Finkelstein (2012)	To review the evidence regarding: (1) Outcomes of health information technology (HIT) applications that address components of patient-centered care (PCC); (2) identifying barriers and facilitators to implementation; (3) defining gaps in our knowledge about these HIT applications that address PCC; and (4) identifying their specific value to consumers, their families, clinicians, and developers of this technology.	Wide heterogeneity of included articles prevented the possibility to perform a meta-analysis. Few studies described the effects of HIT implemented to enable PCC on cost and provider efficiency, and even fewer have done so in a high-quality manner.	Positive effects of PCC-related HIT intervention on: health care process outcomes, disease-specific clinical outcomes (diabetes mellitus, heart disease, cancer), responsiveness to the needs and preferences of patients, shared decision-making, patient-clinician communication, and access to medical information.
Free (2013)	To quantify the effectiveness of mobile technology-based interventions delivered to health care providers or to support health care services, on any health or health care service outcome.	13 trials did not provide sufficient data to calculate effect estimates. Factors influencing heterogeneity of effect estimates included low trial quality and they were not statistically explored. Few trials reporting the same outcomes.	Trials report modest benefits for clinical diagnosis and management support outcomes. SMS[b] reminders have modest benefits on attendance. Service providers should consider implementing SMS appointment reminders.

Georgiou (2013)	To examine evidence of the effect of computerized provider order entry (CPOE) on clinical care and work processes in the ED[c].	Heterogeneous nature of studies, and metrics used to measure performance precluded formal meta-analyses. Potential for publication bias.	Implementation of CPOE systems does not decrease direct patient care time for physicians or nurses, does decrease medication errors, improves laboratory turnaround time, and can influence numbers of laboratory orders but has inconsistent effects on total ED length of stay (LOS). CDSS was shown to consistently improve guideline compliance.
Husebo (2014)	To review the published research on care content and utilization of virtual visits, in particular how old patients and the health care providers use a virtual visit and how they experience it.	The variety of terms used and the interdisciplinary character of the field of home telehealth. Sampling and comparison of heterogeneous studies might lead to biased conclusions.	Virtual visits have the advantages of enhanced social inclusion for the elderly patient and also providing support and guidance in self-management of medication. Virtual visits with ordinary in-person visits could help postpone admission to long-term facilities or the need for substantial in-home care.

Jones (2002)	To examine the impact of telehealth on the clinical nursing of elders and answer two questions: (1) To what extent do telehealth applications support essential components of nursing care of elders? (2) To what extent do telehealth applications support the professional dimensions of gerontological nursing practice?	Due to the preponderance of demonstration and feasibility reports, the dearth of experimental investigations, and the heterogeneous nature of the few studies identified, statistical summarization was not attempted.	Telehealth interventions have the potential to improve nursing care of elders because they provide equivalent approaches to assess physical and psychological state; are acceptable, may prove less costly than face-to-face interventions; and can be delivered in a manner that is timely and convenient.
Kelley (2011)	To examine the relationship between electronic nursing documentation and the quality of care provided to hospitalized patients.	Several studies used descriptive cross-sectional designs, which limit the ability to determine causation. Studies used different instruments to measure nurses' attitudes or perceptions, which limit the comparison of findings.	Research gaps remain across the constructs of quality (structure, process, outcomes), and whether electronic nursing documentation improves quality of care provided to hospitalized patients remains unknown.
Mador (2009)	To provide a comprehensive review of the published empirical literature on the impact of a critical care information system (CCIS) on time spent	Lack of reliance on self-reported or questionnaire-based data and on interrater reliability. No discussion on within- and between-group	It is important that a definition of staff activities be developed and validated so that this can be standardized. The impact of a CCIS on the amount of time that

	documenting and in direct patient care by staff in the Intensive Care Unit (ICU).	differences. All papers were from developed countries and a low number of papers available insert bias.	health care providers (HCPs) spend charting and in direct care remains unclear.
Maenpaa (2009)	To find out how health information systems have been investigated, what has been investigated, and what are the outcomes?	The quality and scope of the analyzed literature. Studies reviewed by one researcher. Difficulty in classifying the studies according to purpose. The review only covered studies in English.	Regional health information system (RHIS) improved clinical data access, timely information, and clinical data exchange and improvement in communication and coordination. There was also inadequate access to patient relevant clinical data.
McKibbon (2011)	To review the evidence on the impact of health information technology (HIT) on all phases of the medication management process.	Literature was heterogeneous; standard definitions are lacking. Majority of research was based on observational methods, often with opportunity for bias. Substantial deficiencies in reporting data.	Nonphysician groups value different aspects of medication management health IT (MMIT), have diverse needs, and use systems differently. Most studies evaluated changes in processes and outcomes of: use, usability, and knowledge, skills, and attitudes. Most showed moderate to substantial improvement with

			implementation of MMIT.
Meissner (2014)	To explore staff experiences within the process of the implementation of computer-based nursing records.	Different settings across studies: this limit the ability to generalize the findings. Timing of data collection was different in all studies, which could mean that experiences may differ.	Staff experience IT as a benefit when it simplifies their daily working routines. When IT complicates them, it is experienced as a burden. The staff experience differs according to duties and responsibilities.
Mickan (2014)	To synthesize high-quality evidence to answer the question: Does health care professionals' use of handheld computers improve their access to information and support clinical decision making at the point of care?	Heterogeneity of studies makes synthesis difficult. There is reason to be concerned about publication bias given the sparse reporting of negative findings.	HCPs use of handheld computers can improve their clinical decision-making through improved information seeking and adherence to clinical guidelines. Handheld computers show promise for real-time access to and analysis of clinical information.
Nguyen (2014)	To review EHR[d] implementations around the world and report findings on benefits and issues associated with EHR implementation.	Lack of well-defined conceptual frameworks for evaluation in various papers. There is a range of sociotechnical theoretical frameworks used in different	The potential of technology to improve clinical documentation quality, increase administration efficiency, lead to better quality, safety, and coordination of care. Negative

		papers.	impacts: changes to workflow and work disruption.
Nieuwlaat (2011)	To determine if CCDSSs improve processes of care or patient outcomes for therapeutic drug monitoring and dosing (TDMD).	Incomplete data for evaluation of the CCDSS effects. Variety of drugs and health care settings were included. These factors combined made it problematic to pool results. Risk for publication bias of positive RCTs, which could cause overestimation of efficacy.	CCDSSs have potential for improving process of care for TDMD. More potent CCDSSs need to be developed, should be evaluated by independent researchers using cluster randomization and primarily assess patient outcomes related to drug efficacy and safety.
Poissant (2005)	To examine the impact of EHRs on documentation time of physicians and nurses and to identify factors that may explain efficiency differences across studies.	Papers included in this review cover a 10-year time period during which technology was rapidly evolving.	Nurses are more likely than physicians to gain time efficiency by using a computer system to document patient information. This could be explained by the fact that nurses and physicians they document different types of information.

Randell (2007)	To examine the effect of CDSSs on nursing performance and patient outcomes.	Heterogeneity in the way the interventions work, protocols on which they are based, and the decision tasks they support. Risk of contamination was a concern in four studies.	The introduction of CDSSs may not lead to a positive outcome; further studies are needed in order to identify contexts in which CDSSs use is most effective. Future studies should seek to explore the significance of each component for nursing performance and patient outcomes.
Stevenson (2010)	To examine nursing documentation in relation to how nurses experience using the EPR[e] in everyday clinical practice in acute or inpatient ward settings.	The small number of studies limits the reliability. There is a limited number of studies on nurses' experience of EPRs in ward settings. Further studies may have been found if the search had been extended to cover a longer period.	Nurses were dissatisfied with EPRs because they did not support everyday clinical practice and were not user-friendly. The nursing documentation software in the EPRs requires a design which is integrated into the clinical workflow and functions optimally in clinical practice.
Urquhart (2009)	To establish the beneficial and adverse effect of nursing record systems on nursing practice and patient outcomes. To establish gaps in knowledge and	Poor methodological quality of studies. Blinded assessment of the outcomes was not reported. Truncation in the presentation of data and results for	The identified studies provide no evidence of any measurable difference, in nursing practice or patient outcomes, between the use of one kind of

	identify areas for further research, both in nursing and in informatics.	publication of all studies has caused some difficulty in assessment.	nursing record system or another.

[a]CDSSs: computerized decision support systems.

[b]SMS: short message service.

[c]ED: emergency department.

[d]EHR: electronic health record.

[e]EPR: electronic personal record.

References

Anderson JA, Willson P. Clinical decision support systems in nursing: synthesis of the science for evidence-based practice. Comput Inform Nurs. 2008;26(3):151-158. PMID: 2009922759

Bartoli L, Zanaboni P, Masella C, Ursini N. Systematic review of telemedicine services for patients affected by chronic obstructive pulmonary disease (COPD). Telemed J E Health. 2009;15(9):877-883. PMID: 19919194

Bowles KH, Baugh AC. Applying research evidence to optimize telehomecare. J Cardiovasc Nurs. 2007;22(1):5-15. PMID: 17224692

Carrington JM, Tiase VL. Nursing informatics year in review. Nurs Adm Q. 2013;37(2):136- 143. PMID: 23454993

Dowding D, Randell R, Gardner P, et al. Dashboards for improving patient care: Review of the literature. Int J Med Inform. 2015 Oct 12;84(2):87-100. PMID: 25453274

Finkelstein J, Knight A, Marinopoulos S, et al. Enabling patient-centered care through health information technology. Rockville, MD: Agency for Healthcare Research and Quality, 2012 Jun. Report No.: 1530-4396. PMID: 24422882

Free C, Phillips G, Watson L, et al. The effectiveness of mobile-health technologies to improve health care service delivery processes: a systematic review and meta-analysis. PLoS Med. 2013;10(1):e1001363. PMID: 23458994

Georgiou A, Prgomet M, Paoloni R, et al. The effect of computerized provider order entry systems on clinical care and work processes in emergency departments: a systematic review of the quantitative literature. Ann Emerg Med. 2013 Jun;61(6):644-653. PMID: 23548404

Husebo AML, Storm M. Virtual visits in home health care for older adults. ScientificWorldJournal. 2014;689873. PMID: 25506616

Jones JF, Brennan PF. Telehealth interventions to improve clinical nursing of elders. Annu Rev Nurs Res. 2002;20:293-322. PMID: 12092513

Kelley TF, Brandon DH, Docherty SL. Electronic nursing documentation as a strategy to improve quality of patient care. J Nurs Scholarsh. 2011 Jun;43(2):154-162. PMID: 21605319

Mador RL, Shaw NT. The impact of a Critical Care Information System (CCIS) on time spent charting and in direct patient care by staff in the ICU: A review of the literature. Int J Med Inform. 2009;78(7):435-445. PMID: 19261544

Maenpaa T, Suominen T, Asikainen P, Maass M, Rostila I. The outcomes of regional healthcare information systems in health care: A review of the research literature. Int J Med Inform. 2009;78(11):757-771. PMID: 19656719

McKibbon KA, Lokker C, Handler SM, et al. Enabling medication management through health

information technology (Health IT). Evidence report/technology assessment: 2011 Apr. Report No.: 1530-4396 PMID: 23126642

Meißner A, Schnepp W. Staff experiences within the implementation of computer-based nursing records in residential aged care facilities: a systematic review and synthesis of qualitative research. BMC Med Inform Decis Mak. 2014;14:54. PMID: 24947420

Mickan S, Atherton H, Roberts NW, Heneghan C, Tilson JK. Use of handheld computers in clinical practice: a systematic review. BMC Med Inform Decis Mak. 2014;14:56. PMID: 24998515

Nguyen L, Bellucci E, Nguyen LT. Electronic health records implementation: An evaluation of information system impact and contingency factors. Int J Med Inform. 2014 Nov;83(11):779-796. PMID: 25085286

Nieuwlaat R, Connolly SJ, Mackay JA, et al. Computerized clinical decision support systems for therapeutic drug monitoring and dosing: a decision-maker-researcher partnership systematic review. Implement Sci. 2011;6:90. PMID: 21824384

Poissant L, Pereira J, Tamblyn R, Kawasumi Y. The impact of electronic health records on time efficiency of physicians and nurses: a systematic review. J Am Med Inform Assoc. 2005 Sep-Oct;12(5):505-516. PMID: 15905487

Randell R, Mitchell N, Dowding D, Cullum N, Thompson C. Effects of computerized decision support systems on nursing performance and patient outcomes: a systematic review. J Health Serv Res Policy. 2007 Oct;12(4):242-249. PMID: 17925077

Stevenson JE, Nilsson GC, Petersson GI, Johansson PE. Nurses'™ experience of using electronic patient records in everyday practice in acute/inpatient ward settings: A literature review. Health Informatics J. 2010;16(1):63-72. PMID: 20413414

Urquhart C, Currell R, Grant MJ, Hardiker NR. Nursing record systems: effects on nursing practice and healthcare outcomes. Cochrane Database Syst Rev. 2009;Art. No.: CD002099(Issue 1). PMID: 19160206
