# Supplementary material for: Impact of Information and Communication Technologies on Nursing Care: Results of an Overview of Systematic Reviews
Source: J Med Internet Res. 2017 Apr 25;19(4):e122. doi: 10.2196/jmir.6686 (PMC5424122; doi:10.2196/jmir.6686)
Supplement: Multimedia Appendix 6 [file jmir_v19i4e122_app6.pdf]

Appendix 6. eHealth domains, interventions, and comparisons.

Review	eHealth domain (intervention)	Type of general and specific ICTs[a]	Examples of included interventions (particularly those used by nurses)	Examples of comparison interventions
Anderson (2008)	CDSSs[b]	Email discussions, handheld computer, pocket preceptor, expert system nursing information system (ES-NIS), PDA[c], telephone advice line with CDSSs, Web-based decision support tool	Examples included clinical decision-making model and novice clinical reasoning model that provides novice nurses with the decision support assistance of an experienced preceptor; wound and skin intelligence system based on clinical practice guidelines; computerized algorithmic decision support; pressure ulcer prevention and management system to assist	In-hospital clinic; APN[d]; nurse eliciting patient preference and usual care

			nurses with individualized guideline-based treatment for patients who have, or are risk for, pressure ulcers.	
Bartoli (2009)	Communi-cation systems	Telemonitoring, telenursing	No specific example	Not specified
Bowles (2007)	Management and Communi-cation systems	Telemanagement, telemonitoring, telecare, telehealth, telemedicine, telehomecare, interactive video technology, electronic health records (EHR)	Examples include physiologic home monitoring and telemanagement on chronic heart failure outcomes; telehomecare to monitor conditions, reinforce patient teaching and compliance, and to support patient and family; heart failure management program through an in-home telehealth communication device; telehealth	Usual care; no intervention; hybrid care (telehomecare and traditional)

			intervention used to reduce secondary conditions among people with spinal cord injury.	
Carrington (2013)	Management systems, communi-cation systems,	Sensor-monitored activity, EHR, electronic medical records (EMR), bedside communication tool, clinical care classification system, continuity of care record, organization risk analyzer (ORA)	Examples include sensors monitoring elderly activity; ORA to explore the relationship of nursing unit communication to patient safety and quality outcomes; interoperable set of diagnosis for use in patient problem list in the EHR to support interoperability; using the clinical care classification	Not specified

			system for costing acute care nursing.	
Dowding (2015)	Management systems	Clinical dashboard, dashboard with EMR, radiology dashboard with picture archiving and communication systems, ventilalor management dashboard, adverse drug event (ADE) dashboard	Examples include ventilator management dashboard, presented as a screensaver and accessible with or without EMR with indicators for each patient for each element of ventilator management bundle	EMR, usual care

Finkelstein (2012)	Management systems, communi-cation systems,	Email, telemedicine, EMR, videophone, video-based home telehealth, clinical reminders	Examples include email reminder highlighting heart failure recommendations, or an augmented intervention of email plus additional prompts, educational material, and outreach by a nurse specialist; smoking advice by nurses and anesthesiologists in a preoperative clinic; clinical decision support system for depression; Web chat for the public to contact a nurse for any kind of health problem.	Usual care, no email, no intervention, nursing patient assessment using paper

Free (2013)	Communi-cation systems; CDSSs	Mobile telephone, PDA, computer-based scoring system, handheld CDSS; portable media player	Examples include enhancing nursing students' pharmacological knowledge during clinical practice; determining whether nursing medication errors could be reduced and nursing care provided more efficiently; comparing the proportion of obesity-related diagnoses in clinical encounters documented by nurses using a PDA-based log with and without obesity decision support feature.	No access to PDA; paper version of same resources; manual scoring and timing; own knowledge and beliefs; textbooks found on unit and calculator; clinical log with height and weight; obesity-related diagnoses from a pick-list of diagnoses for "weight-related condition;" plan of care items from pick-list; paper-based teleforms; consultation of the early warning score (EWS) weightings; usual

				communication procedure
Georgiou (2013)	Management systems, CDSSs	CPOE[e], clinical information systems, nursing documentation system	Examples include effects of implementing CPOE and nursing documentation on provider workflow; effect of implementing clinical information systems on processes of care and outcomes in the ED[f]; integration of clinical decision support into an	Not specified

			existing ED CPOE system; effect of CPOE combined with a bar-code system on the specimen labeling process; effect of CPOE on pediatric ED care providers' allocation of time	
Husebo (2014)	Communi-cation systems	Virtual visits by using videophones and real-time audiovisual communication devices	Examples include virtual visits delivered by videophone were used for observations of the patients' condition, prevention of social isolation and increase in social activities, medication safety, support and monitoring of a chronic medical condition.	Usual care; standard care, videoconference and monitoring; conventional methods on wound home care; telephone service or standard unmonitored medication compliance service.

Jones (2002)	Communi-cation systems	Telehomecare visits, telephone nursing interventions, interactive computer networks, interactive video nursing, interactive video technology, videoconference	Examples include interactive video nursing assessment of cardiac heart failure, wound management using interactive video technology, videoconferencing for the assessment of cognitive functioning	Physical assessments conducted in traditional face-to-face modes
Kelley (2011)	Management systems	EHR, electronic nursing documentation	No specific example	N/A[g]
Mador (2009)	Management systems	Critical care information systems (CCIS)	Electronic medical record (EMR), which is a computerized patient charting system, which has been specifically designed for use in ICU.	Paper-based documentation

Maenpaa (2009)	Management systems	Regional health care information system (RHIS), regional health care information organization (RHIO), disease-specific regional health care information systems (D-RHIS), and integrated regional health care information systems (I-RHIS).	Examples include D-RHIS—exemplary scenario of thyroid disease care in an integrated setting; I-RHIS—electrocardiogram (ECG) or angio processing and management system; Wireless application protocol (WAP)–based system for data transmission from the patient's and from the clinician's side.	Not specified
McKibbon (2011)	Management systems; CDSSs	MMIT[h], e-prescribing applications, electronic medication administration record systems (eMAR), EHRs and EMRs, personal health records (PHRs),	Examples include MMIT applications as electronic systems that (1) collect, process, or exchange health information about patients; (2) are integrated	Not specified.

		health information systems, hospital information systems, CPOE, bar-coded medication administration (BCMA), pharmacy-based HIT, PDAs, patient decision support systems.	with existing health IT systems such as EHR or EMR systems; and (3) provide advice or suggestions on issues or decisions related to medication management.	
Meissner (2014)	Management systems	Computer-based nursing records, clinical information systems, EHR, bedside EMR, electronic documentation	No specific example	N/A
Mickan (2014)	CDSSs	PDA with or without CDSS	Examples include PDA with rule for gastrointestinal risk assessment when prescribing NSAIDS[i]; PDA with angina diagnosis software. PDA with CDSS for	PDA without rule for gastrointestinal risk assessment when prescribing NSAIDS; conventional care; PDA without CDSS for obesity

			obesity diagnosis; PDA with CDSS for pulmonary embolism.	diagnosis; PDA used for data collection only; paper-based guideline material.
Nguyen (2014)	Management systems	EHR	No specific example	N/A
Nieuwlaat (2011)	CDSSs	CDSSs used for TDMD	CDSSs (computer-assisted insulin protocol) recommended insulin dosing and glucose monitoring to achieve glucose control in patients in intensive care units.	A strict glycemic control protocol for intravenous insulin infusion. All insulin adjustments were made by nurses. Conventional and usual care: Subcutaneous insulin is administered for blood glucose levels.
Poissant (2005)	Management systems	EHR, central station desktops such as computerized	The PDA was used to enter data on an activity of daily living	Paper charting

		nursing documentation system, PDA, bedside terminals	(ADL) assessment tool and was used as an independent device with no data exchange at the time of data entry. IT were used for documentation (ie, all notes, orders, and referrals that are part of the care plan of a patient and documented in a patient’s medical chart).	
Randell (2007)	CDSSs	CDSSs	Warfarin dosage adjustment calculated using CDSSs; glucose regulation by ICU nurses using CDSSs; telephone triage and advice with CDSSs	Warfarin dosage adjustment calculated by nurse-specialist without CDSSs; patients requesting same day appointment triaged by practice nurses

				supported by clinical protocols; Glucose regulation by ICU nurses using paper-based guidelines; standard care
Stevenson (2010)	Management systems	EPR	Examples include computerized patient information systems (CPIS) for clinical information; EHRs for documentation in nursing	Not specified
Urquhart (2009)	Management systems	Nursing record systems	Examples included computerized care-planning system incorporating standardized nursing nomenclature, the system was used for recording care as well as for	Paper-based documentation system, paper-based care plans, paper nursing care planning system

			care planning; intensive care information system	

[a]ICTs: information and communication technologies.

[b]CDSSs: computerized decision support systems.

[c]PDA: personal digital assistant.

[d]APN: advanced practice nurse.

[e]CPOE: computerized provider order entry.

[f]ED: emergency department.

[g]N/A: not applicable.

[h]MMIT: medication management health information technology

[i]NSAIDS: nonsteroidal antiinflammatory drugs.

References

Anderson JA, Willson P. Clinical decision support systems in nursing: synthesis of the science for evidence-based practice. Comput Inform Nurs. 2008;26(3):151-158. PMID: 2009922759

Bartoli L, Zanaboni P, Masella C, Ursini N. Systematic review of telemedicine services for patients affected by chronic obstructive pulmonary disease (COPD). Telemed J E Health. 2009;15(9):877-883. PMID: 19919194

Bowles KH, Baugh AC. Applying research evidence to optimize telehomecare. J Cardiovasc Nurs. 2007;22(1):5-15. PMID: 17224692

Carrington JM, Tiase VL. Nursing informatics year in review. Nurs Adm Q. 2013;37(2):136-143. PMID: 23454993

Dowding D, Randell R, Gardner P, et al. Dashboards for improving patient care: Review of the literature. Int J Med Inform. 2015 Oct 12;84(2):87-100. PMID: 25453274

Finkelstein J, Knight A, Marinopoulos S, et al. Enabling patient-centered care through health information technology. Rockville, MD: Agency for Healthcare Research and Quality, 2012 Jun. Report No.: 1530-4396. PMID: 24422882

Free C, Phillips G, Watson L, et al. The effectiveness of mobile-health technologies to improve health care service delivery processes: a systematic review and meta-analysis. PLoS Med. 2013;10(1):e1001363. PMID: 23458994

Georgiou A, Prgomet M, Paoloni R, et al. The effect of computerized provider order entry systems on clinical care and work processes in emergency departments: a systematic review of the quantitative literature. Ann Emerg Med. 2013 Jun;61(6):644-653. PMID: 23548404

Husebo AML, Storm M. Virtual visits in home health care for older adults. ScientificWorldJournal. 2014;689873. PMID: 25506616

Jones JF, Brennan PF. Telehealth interventions to improve clinical nursing of elders. Annu Rev Nurs Res. 2002;20:293-322. PMID: 12092513

Kelley TF, Brandon DH, Docherty SL. Electronic nursing documentation as a strategy to improve quality of patient care. J Nurs Scholarsh. 2011 Jun;43(2):154-162. PMID: 21605319

Mador RL, Shaw NT. The impact of a Critical Care Information System (CCIS) on time spent charting and in direct patient care by staff in the ICU: A review of the literature. Int J Med Inform. 2009;78(7):435-445. PMID: 19261544

Maenpaa T, Suominen T, Asikainen P, Maass M, Rostila I. The outcomes of regional healthcare information systems in health care: A review of the research literature. Int J Med Inform. 2009;78(11):757-771. PMID: 19656719

McKibbon KA, Lokker C, Handler SM, et al. Enabling medication management through health information technology (Health IT). Evidence report/technology assessment: 2011 Apr. Report No.: 1530-4396 PMID: 23126642

Meißner A, Schnepp W. Staff experiences within the implementation of computer-based nursing records in residential aged care facilities: a systematic review and synthesis of qualitative research. BMC Med Inform Decis Mak. 2014;14:54. PMID: 24947420

Mickan S, Atherton H, Roberts NW, Heneghan C, Tilson JK. Use of handheld computers in clinical practice: a systematic review. BMC Med Inform Decis Mak. 2014;14:56. PMID: 24998515

Nguyen L, Bellucci E, Nguyen LT. Electronic health records implementation: An evaluation of information system impact and contingency factors. Int J Med Inform. 2014 Nov;83(11):779-796. PMID: 25085286

Nieuwlaat R, Connolly SJ, Mackay JA, et al. Computerized clinical decision support systems for therapeutic drug monitoring and dosing: a decision-maker-researcher partnership systematic review. Implement Sci. 2011;6:90. PMID: 21824384

Poissant L, Pereira J, Tamblyn R, Kawasumi Y. The impact of electronic health records on time efficiency of physicians and nurses: a systematic review. J Am Med Inform Assoc. 2005 Sep-Oct;12(5):505-516. PMID: 15905487

Randell R, Mitchell N, Dowding D, Cullum N, Thompson C. Effects of computerized decision support systems on nursing performance and patient outcomes: a systematic review. J Health Serv Res Policy. 2007 Oct;12(4):242-249. PMID: 17925077

Stevenson JE, Nilsson GC, Petersson GI, Johansson PE. Nurses'™ experience of using electronic patient records in everyday practice in acute/inpatient ward settings: A literature review. Health Informatics J. 2010;16(1):63-72. PMID: 20413414

Urquhart C, Currell R, Grant MJ, Hardiker NR. Nursing record systems: effects on nursing practice and healthcare outcomes. Cochrane Database Syst Rev. 2009;Art. No.: CD002099(Issue 1). PMID: 19160206
